# Optical Spectroscopy and Spectral Imaging for Bladder Cancer Detection and Characterisation: A Scoping Review of Diagnostic Evidence and Translational Readiness

**DOI:** 10.3390/diagnostics16162561

**Published:** 2026-08-14

**Authors:** Arthur Vander Stichele, Ben Van Cleynenbreugel

**Affiliations:** 1 Faculty of Medicine, KU Leuven, Herestraat 49—box 400, 3000 Leuven, Belgium; ben.vancleynenbreugel@uzleuven.be; 2 Department of Urology, University Hospitals Leuven (UZ Leuven), Herestraat 49, 3000 Leuven, Belgium

**Keywords:** bladder cancer, urothelial carcinoma, optical spectroscopy, Raman spectroscopy, hyperspectral imaging, multispectral imaging, optical diagnostics, cystoscopy, tissue characterisation, scoping review

## Abstract

**Background/Objectives:** White-light cystoscopy remains central to bladder cancer detection and surveillance, but diagnostic uncertainty persists in detecting flat lesions, particularly carcinoma in situ, distinguishing inflammatory mimics, and targeting biopsies. Optical spectroscopy and spectral imaging may provide objective tissue information beyond visual inspection. This scoping review mapped the diagnostic evidence and translational readiness of these technologies. **Methods:** A scoping review was performed in accordance with the Preferred Reporting Items for Systematic Reviews and Meta-Analyses extension for Scoping Reviews (PRISMA-ScR). PubMed, Embase and Web of Science were searched from inception to 31 July 2026. Eligible articles evaluated wavelength-dependent optical signals directly in human bladder tissue in clinically relevant in vivo or ex vivo settings. Related articles with the same, overlapping or expanded datasets were grouped into study families. **Results:** Forty-seven articles were organised into 39 study families. Raman spectroscopy showed the clearest progression from ex vivo discrimination to in vivo cystoscopic feasibility and device development. Fluorescence and reflectance provided early in vivo evidence, although specificity was often limited by inflammation and benign changes. Infrared and near-infrared techniques remained mainly specimen-based, while multimodal systems were evaluated mostly ex vivo. Evidence for spectral imaging was limited, and no clinically integrated endoscopic hyperspectral-imaging study meeting the criteria was identified. **Conclusions:** Optical spectroscopy and spectral imaging show diagnostic potential for bladder cancer tissue characterisation, but evidence remains heterogeneous and insufficient for routine implementation. Among the included techniques, Raman spectroscopy appears most translationally mature, whereas wide-field spectral imaging remains underdeveloped. Future studies should prioritise prospective in vivo evaluation, standardised acquisition, histopathological correlation, external patient-level validation and clinically meaningful endpoints.

## 1. Introduction

Bladder cancer remains a common urological malignancy and continues to impose a substantial clinical burden worldwide, with approximately 635,000 new cases and 228,000 deaths estimated in 2024 [1]. Approximately 70–75% of patients present with non-muscle-invasive bladder cancer (NMIBC), which is characterised by high recurrence rates and a risk of progression, necessitating long-term cystoscopic surveillance [2].

White-light cystoscopy (WLC) remains the standard method for the detection and surveillance of bladder tumours. However, its diagnostic performance is limited in several clinically relevant scenarios, particularly in the detection of flat or subtle lesions such as carcinoma in situ (CIS). In addition, WLC may have limited specificity in distinguishing malignant lesions from benign inflammatory, reactive or treatment-related urothelial changes. Adjunctive techniques such as blue-light cystoscopy (BLC) and narrow-band imaging (NBI) can improve lesion detection, but false-positive findings and imperfect specificity remain important limitations [2,3].

In daily clinical practice, these diagnostic challenges frequently result in uncertainty, particularly in patients with positive or suspicious urinary cytology in the absence of a clearly visible lesion on WLC. In such cases, random or mapping bladder biopsies may be performed to exclude occult CIS, despite these being invasive procedures with variable diagnostic yield. This clinical scenario highlights the need for technologies that can provide more objective tissue information during cystoscopic evaluation [4].

Against this background, optical spectroscopy and related spectral techniques have attracted increasing interest as a way to assess bladder tissue beyond conventional visual inspection. By analysing tissue–light interactions across one or more wavelength ranges, these techniques aim to capture biochemical, structural and optical features that are not visible under standard white light. Imaging-based approaches such as multispectral imaging (MSI) and hyperspectral imaging (HSI) extend this principle across a field of view. By contrast, probe-based spectroscopy provides local tissue characterisation and may function as an optical biopsy [5,6].

Although conventional BLC/PDD and NBI use selected wavelength ranges, they are established image-enhancement techniques rather than methods for objective spectral tissue characterisation. Standalone optical coherence tomography (OCT) is primarily structural. The present review therefore focuses on spectroscopy and dedicated spectral imaging, while retaining hybrid systems with a separately interpretable spectroscopic component [2,3].

Previous reviews have addressed advanced optical diagnosis in bladder cancer more broadly or have focused specifically on vibrational spectroscopy [3,7]. The present review maps Raman, fluorescence, reflectance, infrared, multimodal and dedicated spectral-imaging approaches within a single clinically oriented framework.

Technical feasibility alone does not establish diagnostic utility. For clinical translation, these technologies must demonstrate added value in tasks such as lesion detection, biopsy targeting, differentiation of malignancy from inflammatory changes, grading, staging, or intraoperative tissue characterisation.

Therefore, we conducted a scoping review to examine the current literature on optical spectroscopy and spectral imaging for bladder cancer detection and characterisation. The objectives were to map the available evidence, compare technology families, assess translational readiness, and identify the technical and clinical gaps that should guide future studies.

## 2. Methods

### 2.1. Study Design

This scoping review was designed to map the available literature on optical spectroscopy and related spectral techniques for bladder cancer detection and characterisation. The scope included point-based spectroscopy, spectroscopic mapping and imaging-based approaches such as MSI or HSI.

A scoping review was considered appropriate because the field remains emerging and heterogeneous. The available studies differ widely in technical platform, clinical setting, analytical method and reported outcomes. The aim was therefore to provide a structured overview of the technologies studied, their diagnostic potential, and the main barriers to clinical translation rather than to perform a meta-analysis.

The review was conducted in accordance with the Preferred Reporting Items for Systematic Reviews and Meta-Analyses extension for Scoping Reviews (PRISMA-ScR) [8]. The methodological approach was informed by updated guidance for the conduct, extraction, analysis and presentation of scoping reviews [9,10]. No protocol was prospectively registered. A completed PRISMA-ScR checklist has been submitted as Supplementary Material (Supplementary File S1: PRISMA-ScR checklist).

During revision, the search strategy was broadened to improve retrieval across the full range of eligible techniques, and the eligibility criteria were clarified to support consistent study selection. In particular, the terminology for Fourier-transform infrared spectroscopy (FTIR), attenuated total reflectance infrared spectroscopy (ATR-IR), spectroscopic imaging and related tissue-based methods was expanded. All databases were then rerun from inception to 31 July 2026 using the final strategy.

### 2.2. Literature Search Strategy

A structured literature search was performed in PubMed/MEDLINE, Embase and Web of Science Core Collection from database inception to 31 July 2026.

The search combined terms for bladder cancer, urothelial carcinoma and bladder tissue with terms for spectroscopy and spectral imaging. The latter covered Raman, fluorescence and autofluorescence, diffuse reflectance, infrared and near-infrared spectroscopy, and multispectral or hyperspectral imaging. Controlled vocabulary was combined with free-text keywords according to the syntax of each database. The complete search strategies are provided in Supplementary File S2.

The final searches retrieved 1063 records from PubMed, 1616 from Embase and 1659 from Web of Science. After automated and manual duplicate removal, 2501 unique records remained. Targeted backward citation searching involved reviewing reference lists of included articles and relevant reviews, while forward citation searching examined later articles citing key included studies. One additional report entered the full-text pathway through these searches.

### 2.3. Eligibility Criteria

Eligibility criteria were defined according to the Population–Concept–Context framework.

**Population.** Studies involving patients with suspected or confirmed bladder cancer, or direct analysis of human bladder tissue obtained during cystoscopy, biopsy, transurethral resection of bladder tumour (TURBT), cystectomy or a comparable clinical procedure, were eligible. Mixed-organ studies were included only when bladder-specific findings were separately identifiable. Urothelial series that were otherwise bladder-focused were retained when they contained an isolated upper-tract specimen, provided that bladder tissue clearly predominated. This limitation was reported. Technical companion articles were retained when they provided additional acquisition or platform information for an otherwise eligible bladder-specific study family.

**Concept.** Studies were eligible if they acquired and analysed wavelength-dependent optical signals directly from human bladder tissue for tumour detection, tissue characterisation, CIS detection, grading, staging, biopsy guidance, tissue mapping, or technical and clinical feasibility. Eligible approaches included Raman spectroscopy and imaging, fluorescence or autofluorescence spectroscopy, diffuse reflectance or elastic scattering, infrared and near-infrared spectroscopy, dedicated MSI or HSI, wavelength-resolved nonlinear imaging, and multimodal systems with a separately interpretable spectral component. Studies using exogenous contrast agents were included only when spectral measurements were central to the analysis.

**Context.** Both in vivo cystoscopic or intraoperative studies and ex vivo studies of intact or bulk human bladder specimens were included. Studies using unstained tissue sections were eligible when they directly characterised bladder tissue rather than forming part of a digital-pathology workflow. Fresh tissue was considered most relevant to clinical translation. Frozen, fixed, dried or otherwise processed tissue was retained as earlier-stage evidence.

### 2.4. Exclusion Criteria

Studies were excluded when they did not directly assess human bladder tissue or did not provide separately extractable bladder-specific findings. This included animal, phantom, cell-line, organoid, isolated-cell and biofluid studies.

Studies were also excluded if they evaluated visual image enhancement without spectral analysis, such as conventional PDD, NBI, red-green-blue (RGB) colour ratios, single-band targeted fluorescence or standalone structural OCT. We also excluded studies focused solely on digital pathology or thin-section microscopy without direct endoscopic or specimen-based relevance, and multiphoton or fluorescence lifetime imaging microscopy (FLIM) studies without wavelength-resolved spectra. Conference abstracts, reviews, non-peer-reviewed reports and non-English full texts were excluded.

### 2.5. Study Selection

All identified records were managed in EndNote 20 (Clarivate Analytics, Philadelphia, PA, USA). Duplicate records were removed automatically using the digital object identifier (DOI), title, author and publication year, followed by manual checking.

**Figure 1:**
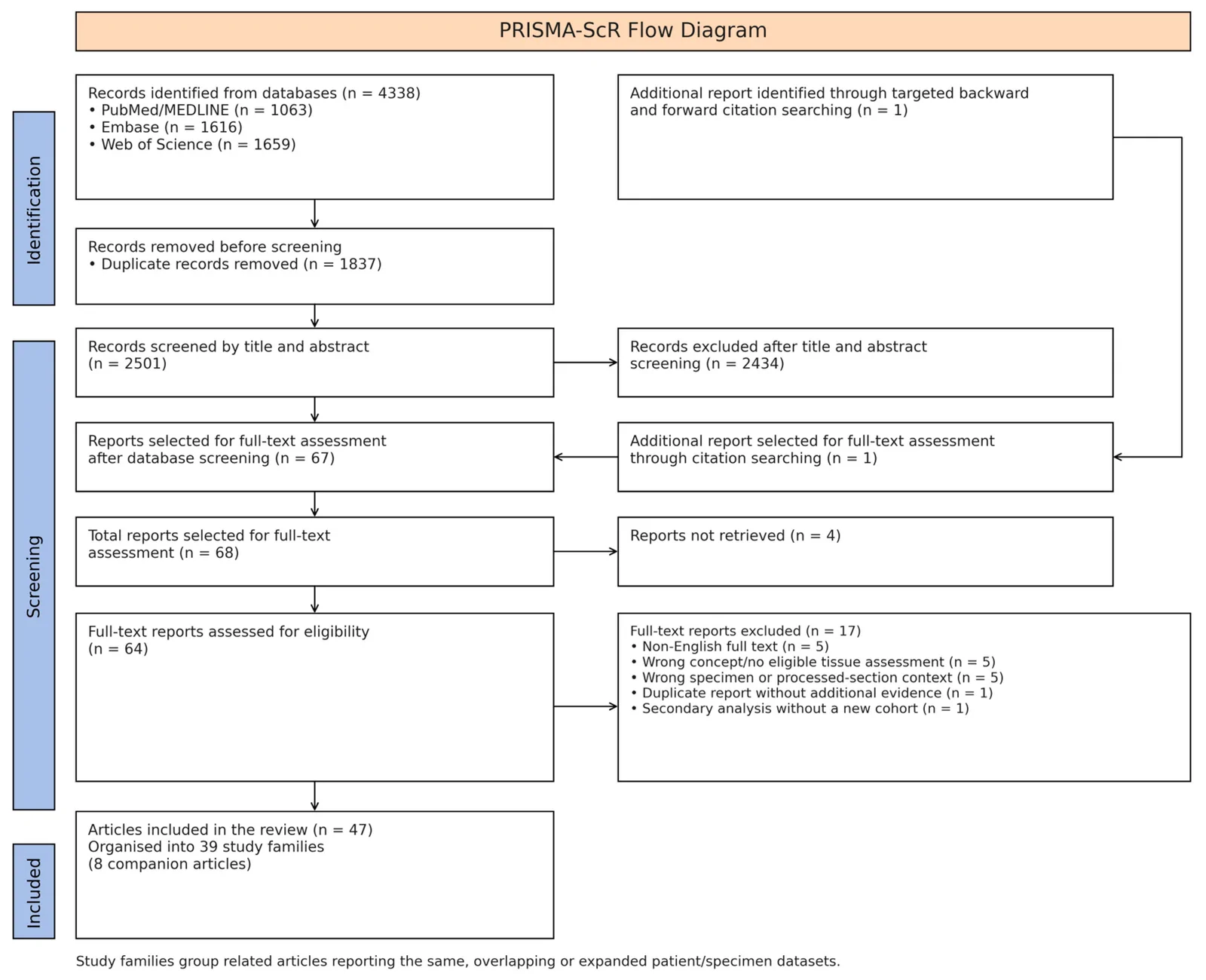
Preferred Reporting Items for Systematic Reviews and Meta-Analyses extension for Scoping Reviews (PRISMA-ScR) flow diagram showing study identification, screening, eligibility assessment and inclusion. Adapted from the PRISMA 2020 flow diagram by Page et al. [11], licenced under the Creative Commons Attribution 4.0 International Licence.

The first author screened titles, abstracts and full texts. Uncertain eligibility decisions were discussed with the senior author. Records identified by the updated searches, together with previously excluded records that became potentially eligible after clarification of the criteria or citation checking, were reassessed against the final eligibility criteria. During revision, the senior author reviewed and approved the final scope and study-family framework and independently cross-checked a targeted sample of key extracted results against the source publications. The study-selection process is shown in Figure 1.

### 2.6. Data Extraction and Study-Family Mapping

Data extraction was performed by the first author using a standardised charting form. Extracted variables included study design, patient and specimen numbers, tissue source and preparation, clinical setting, investigated technology, spectral range, analytical method, diagnostic task, validation approach, reported performance, proportion of usable measurements, and the main limitations.

A study family comprised one or more related articles reporting the same, overlapping or expanded patient or specimen dataset. The most complete article was treated as the main article, while additional technical, preliminary or interpretive publications were classified as companion articles. Separate cohorts reported within a single article were charted separately. When a later article combined a new cohort with a reused comparator, it was assigned to the new cohort’s family; the reused component was linked to its source study and was not counted again.

### 2.7. Synthesis and Methodological Interpretation

Because of substantial differences in acquisition systems, tissue preparation, study populations, analytical methods and reported outcomes, the findings were synthesised descriptively. No quantitative pooling or meta-analysis was performed.

The evidence was organised into four groups: Raman spectroscopy; fluorescence, reflectance and infrared/near-infrared spectroscopy; multimodal or hybrid systems; and spectral imaging. In vivo and ex vivo findings were interpreted separately where clinically relevant. Translational readiness was assessed narratively according to tissue context, in vivo use, acquisition speed, the proportion of usable measurements, patient-level validation, histopathological correlation, and integration into the cystoscopic or perioperative workflow.

The included evidence ranged from descriptive spectral and mapping studies to feasibility studies and diagnostic classifiers. Because no single formal risk-of-bias tool was suitable for all of these designs, no formal instrument was applied. Instead, sample size, histopathological correlation, validation approach, repeated measurements, related articles and clinical applicability were considered when interpreting the findings.

### 2.8. Use of Artificial Intelligence Tools

Generative artificial intelligence was used to support search-term refinement, screening triage, structured extraction and organisation of study information, identification of potentially related reports, consistency checking, preparation of draft tables and schematic figures, and language editing. It was not treated as an independent reviewer and did not make final eligibility or interpretive decisions. Final decisions were made by the authors; the first author checked the reported data against the source publications, with targeted independent cross-checking by the senior author. Product details are provided in the acknowledgments.

## 3. Results

### 3.1. Study Characteristics

Forty-seven articles met the inclusion criteria and were organised into 39 study families. Eight were companion articles reporting the same, overlapping or expanded datasets. Study families were used to organise related reports and should not be interpreted as fully independent patient cohorts. The included literature was published between 1993 and 2024.

Study designs ranged from in vivo cystoscopic or intraoperative investigations to ex vivo analyses of fresh, frozen, fixed or otherwise processed bladder tissue. Sixteen study families included in vivo measurements, 22 were performed exclusively ex vivo, and one combined in vivo and ex vivo measurements. Most were small feasibility or proof-of-concept studies rather than large prospective clinical validation studies.

Clinically, it is important to distinguish between in vivo and ex vivo evidence. Ex vivo studies show what a technology can achieve under controlled conditions. In vivo cystoscopy introduces additional sources of variation, including bladder filling and wall movement, irrigation, urine or blood, tissue curvature, and differences in probe contact or distance across bladder sites.

**Figure 2:**
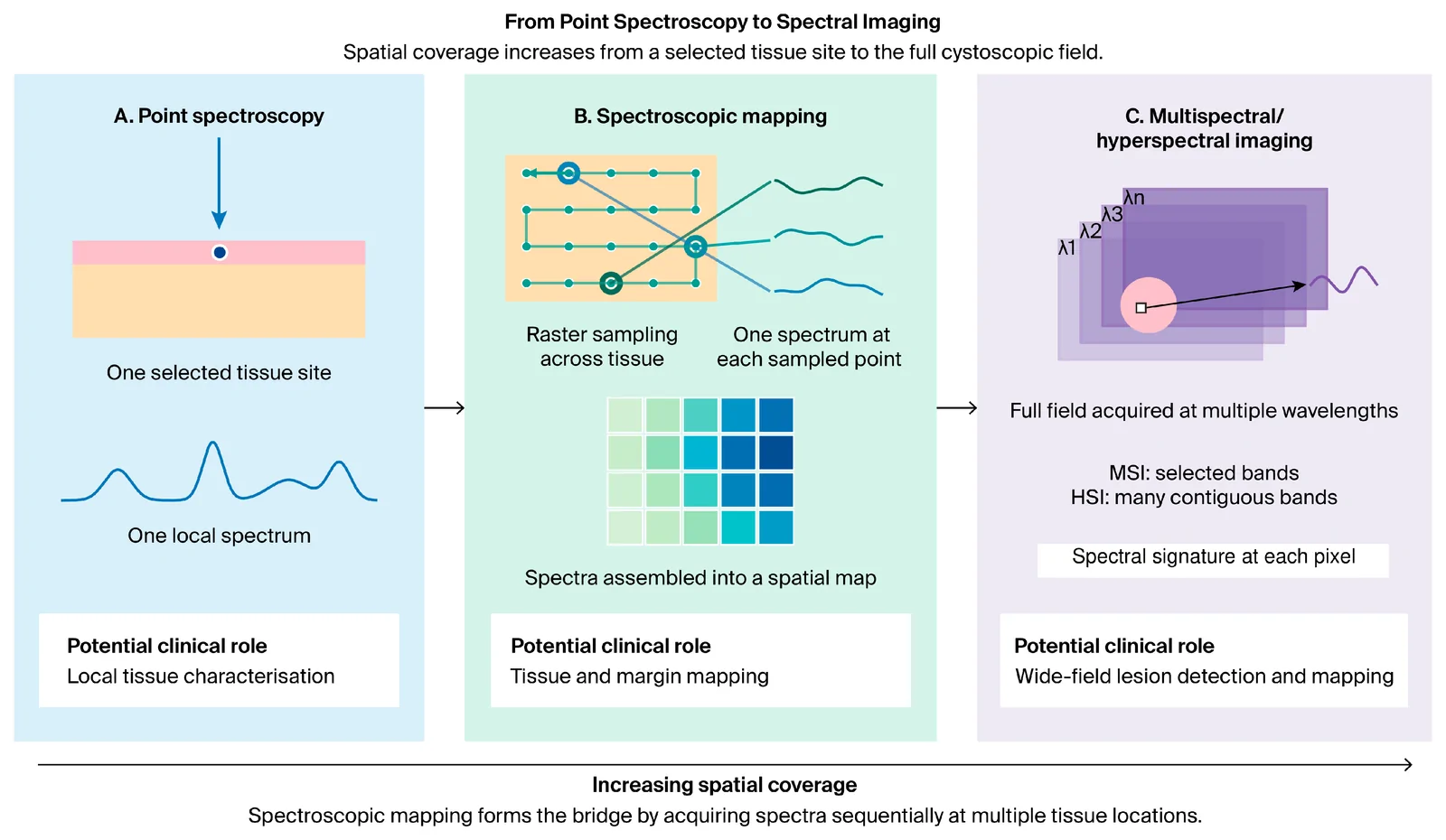
Relationship between point spectroscopy, spectroscopic mapping, multispectral imaging (MSI) and hyperspectral imaging (HSI). The principal distinction is spatial coverage, although the categories overlap when spectra are acquired at multiple tissue locations. Original conceptual illustration. (The colours are used only to distinguish the three conceptual approaches and do not encode quantitative information.)

The included study families are summarised in Table 1. Additional article-level characteristics, tissue preparation, validation methods and key performance data are provided in Supplementary Tables S1 and S2. Point spectroscopy provides a spectrum from one selected tissue site. Spectroscopic mapping acquires spectra sequentially at multiple locations and assembles them into a spatial map, whereas MSI or HSI captures spectral information across an image field and provides a spectral signature for each pixel. Spectroscopic mapping therefore forms a conceptual bridge between local spectroscopy and wide-field spectral imaging (Figure 2).

### 3.2. Raman Spectroscopy

#### 3.2.1. Probe-Based Raman Spectroscopy

Raman spectroscopy was the most extensively studied single technology. Early ex vivo studies showed reproducible spectral differences related to proteins, lipids, nucleic acids, collagen, glycogen and other molecular components. They reported discrimination between normal tissue, cystitis, CIS and tumours, with additional exploration of grade and stage. However, several analyses relied on many spectra from relatively few patients or specimens [12,13,14,15,16,17,19,20,21,39].

Probe design influenced which tissue layers were sampled. High-volume probes collected signal from a larger and deeper tissue volume, whereas confocal or superficial probes focused more strongly on the urothelium. This may reduce signal dilution from deeper connective or muscle tissue, but it makes consistent probe position and contact more important. Draga et al. demonstrated in vivo acquisition, while Stomp-Agenant et al. later evaluated a superficial probe in 66 new patients and used the 38-patient Draga cohort as a comparator. The superficial probe achieved 90% sensitivity, 87% specificity and an area under the curve (AUC) of 0.95 using leave-one-patient-out analysis, although inflammation remained an important source of error [18,19,24].

More recent work focused on clinical integration. Latka et al. developed a Raman system within the European medical-device framework. Only 13 of 21 patients yielded spectra suitable for analysis, and 438 of 800 acquired spectra (55%) were retained. The reported 83% accuracy came from internal cross-validation of these retained spectra, and whether spectra were kept together by patient was not reported. Tissue fluorescence, endoscope illumination and Hexvix interference accounted for much of the data loss. The result therefore applies only to the selected usable spectra, while the high failure rate remains an important obstacle to routine use [28].

Liu et al. reported a diagnostic cohort and a separate 53-patient treatment-comparison cohort. Based on the reported confusion matrix, cancer sensitivity and specificity were 80.8% and 90.5%, respectively; these values differed from the labels stated in the article. The diagnostic estimates were based on leaving out one spectrum at a time rather than on patient-level validation. In the treatment comparison, only 7 of 27 patients in the Raman-guided group received cryoablation, and recurrence occurred in 2/27 versus 8/26 controls. Because randomisation details, power, histological confirmation of Raman-positive residual tumour and time-to-event analysis were not reported, this result should be viewed as exploratory evidence for a combined Raman-guided treatment pathway, not proof that Raman diagnosis itself reduces recurrence [27].

Overall, probe-based Raman spectroscopy has progressed furthest from laboratory analysis towards direct cystoscopic use. Nevertheless, signal quality, inflammation, probe access, sampling depth and limited external validation continue to restrict clinical interpretation.

#### 3.2.2. Raman Spectroscopic Imaging and Surface-Enhanced Raman Scattering

Raman spectroscopic imaging collects spectra across many tissue points to create a spatial map. Ex vivo studies identified tumour, normal tissue, necrosis and other tissue or material components within resected specimens. This spatial information reveals tissue heterogeneity, but acquisition and analysis are slower than point measurements, and the available studies remained small and ex vivo [16,25,26].

One small study used surface-enhanced Raman scattering (SERS), in which topically applied nanoparticles amplified signals from selected molecular targets. Unlike the measurements used in most Raman studies, SERS depends on an exogenous contrast agent. It showed promising molecular contrast in resected tissue but was evaluated in only 15 specimens used for classification and remained at an early experimental stage [22].

### 3.3. Fluorescence, Reflectance, Infrared and Near-Infrared Spectroscopy

Fluorescence and reflectance spectroscopy provided some of the earliest in vivo evidence in this field. Studies evaluated Photofrin-enhanced fluorescence, native autofluorescence, elastic light scattering and diffuse reflectance during cystoscopy or immediately before biopsy. Several studies reported strong separation between malignant and benign tissue, but many cut-offs were developed and tested in the same small datasets [29,30,31,32,33,34,35,36,37].

The Koenig programme illustrated both the promise and the limitations of autofluorescence-guided biopsy. In the expanded 1998 study, a threshold retaining 95% of malignant lesions could theoretically have reduced biopsies from non-malignant tissue by 72%. However, 44 suspicious lesions had already been included in the 1996 companion article. The two reports were therefore treated as one study family: their patient and lesion counts were not added, and the later results were not interpreted as independent confirmation of performance [32,33].

Later studies confirmed measurable fluorescence differences between benign and malignant tissue. Aboumarzouk et al. identified several discriminatory spectral variables but did not validate a classification rule. Palmer et al. found that an autofluorescence-based measure of tissue metabolism separated CIS from healthy tissue better than it separated the overall cancer group from healthy tissue (exploratory AUC 0.83 versus 0.64). However, the CIS estimate was based on only six samples and was not independently validated. In a wide-field autofluorescence pilot, Kriegmair et al. reported high sensitivity but low specificity, while the separate spectroscopy sub-study involved only six patients [40,41,42].

Infrared and near-infrared spectroscopy were studied mainly ex vivo. FTIR and ATR-IR studies showed cancer-associated differences in molecular absorption patterns linked to proteins, lipids, nucleic acids and collagen, although several used fixed, dried or powdered tissue and did not develop validated diagnostic models. More recent fresh-tissue studies have reported promising results for tumour detection and grade or stage discrimination and may be most relevant for rapid assessment of resected tissue after TURBT. However, this role has not been tested in a prospective clinical workflow, and validation often used multiple spectra per specimen rather than independent patients, which limits the generalisability of the reported performance [38,39,44,45,46,47].

Taken together, these approaches show that bladder cancer produces measurable optical and molecular changes. Their reported performance should, however, be interpreted in relation to tissue preparation, study size, benign comparators and validation method.

### 3.4. Multimodal and Hybrid Systems

Multimodal systems combine signals that describe different aspects of tissue. Raman spectroscopy provides molecular information, fluorescence reflects naturally occurring tissue fluorescence or administered photosensitisers, diffuse reflectance shows absorption and scattering, and OCT adds structural detail.

Small OCT–Raman studies suggested that structural and molecular information could complement each other. Bovenkamp et al. included only eight patients. Placzek et al. analysed 44 patients and 119 biopsies, while Schie et al. used the same cohort for a companion analysis of how tissue composition influenced OCT appearance. All of these studies remained ex vivo [48,49,50].

A separate multimodal fibre-probe programme combined fluorescence, Raman and reflectance spectroscopy. Baria et al. reported preliminary findings, and Morselli et al. later described the expanded 114-patient programme. The approach supported tumour detection, grade and stage discrimination, but it was evaluated on resected specimens with internal validation [51,52].

Zlobina et al. provided an in vivo comparison of several spectral combinations in 21 patients. Diffuse reflectance plus visible fluorescence performed best, while adding near-infrared fluorescence and Raman features did not improve classification. This illustrates that more signals do not automatically produce a better clinical test [53].

Overall, multimodal and hybrid systems are promising, but the current evidence remains limited by small cohorts, mainly ex vivo designs, additional system complexity and uncertain added value over simpler combinations.

### 3.5. Spectral Imaging Techniques

Spectral imaging is clinically attractive because cystoscopy requires inspection of the entire bladder surface rather than measurement of one point alone. Imaging systems may therefore help locate subtle or flat lesions, after which point spectroscopy could provide more detailed local characterisation.

Demos et al. provided an early ex vivo example of spectral imaging in fresh resected bladder tissue. The method combined near-infrared autofluorescence with a scattering-based image to highlight malignant areas and visually outline tumour margins. Although it combined two optical signals, it was grouped with spectral imaging because its main output was a spatial image rather than a point measurement. Jacobson et al. later showed that simultaneous white-light and near-infrared autofluorescence imaging was feasible during cystoscopy. Kriegmair et al. evaluated real-time multispectral cystoscopy in ten patients. All malignant lesions were recognised by both observers on the multiparametric images, but this was a selected feasibility study rather than a formal diagnostic-accuracy trial [54,56,58].

Two-photon spectral microscopy was explored in small ex vivo cohorts and showed differences related to CIS and tumour grade. Because it required microscopic analysis of excised specimens, it was not tested as a real-time cystoscopic diagnostic tool [55,57].

No clinically integrated endoscopic HSI study meeting the eligibility criteria was identified. The available human imaging evidence instead consisted mainly of near-infrared spectral imaging, real-time multispectral imaging and small specimen-based spectral microscopy studies. HSI therefore remains an important research opportunity rather than an established clinical evidence track in bladder cancer [5,6,58].

### 3.6. Overall Trends Across the Included Studies

Overall, the literature shows a gradual progression from early optical proof-of-concept studies towards more clinically integrated spectroscopy systems. Raman spectroscopy showed the clearest movement towards in vivo use, whereas fluorescence and reflectance had a longer but less consistent in vivo history. Infrared, multimodal and imaging-based techniques remained predominantly ex vivo or feasibility-based.

Direct comparison between studies was difficult because patient numbers, tissue preparation, acquisition systems, diagnostic targets and analytical methods varied widely. Several articles also arose from the same patient or specimen series. Grouping related articles into study families reduced obvious double counting and showed that publication counts alone overstate the amount of independent evidence.

Most importantly, strong reported accuracy in a controlled specimen study did not necessarily indicate readiness for cystoscopic use. Only two study families clearly used patient-grouped internal validation, and none used an independent external cohort. Clinically realistic benign comparators and transparent reporting of unusable measurements also remained uncommon.

## 4. Discussion

### 4.1. Principal Findings

This scoping review describes a field with real promise, but not yet a clinically mature evidence base. Forty-seven articles were organised into 39 study families after related articles reporting the same, overlapping or expanded datasets were linked. Raman spectroscopy stood out as the most developed line of research, with repeated ex vivo studies, several in vivo cohorts, and probe and device-development work within the European medical-device framework. Fluorescence and reflectance spectroscopy provided an important historical and clinical foundation, while infrared and near-infrared methods remained mainly specimen-based. Multimodal systems showed complementary information but were evaluated mostly ex vivo. Clinically integrated wide-field spectral imaging, especially HSI, remained comparatively underdeveloped.

### 4.2. Biological Basis of Spectral Differences

**Figure 3:**
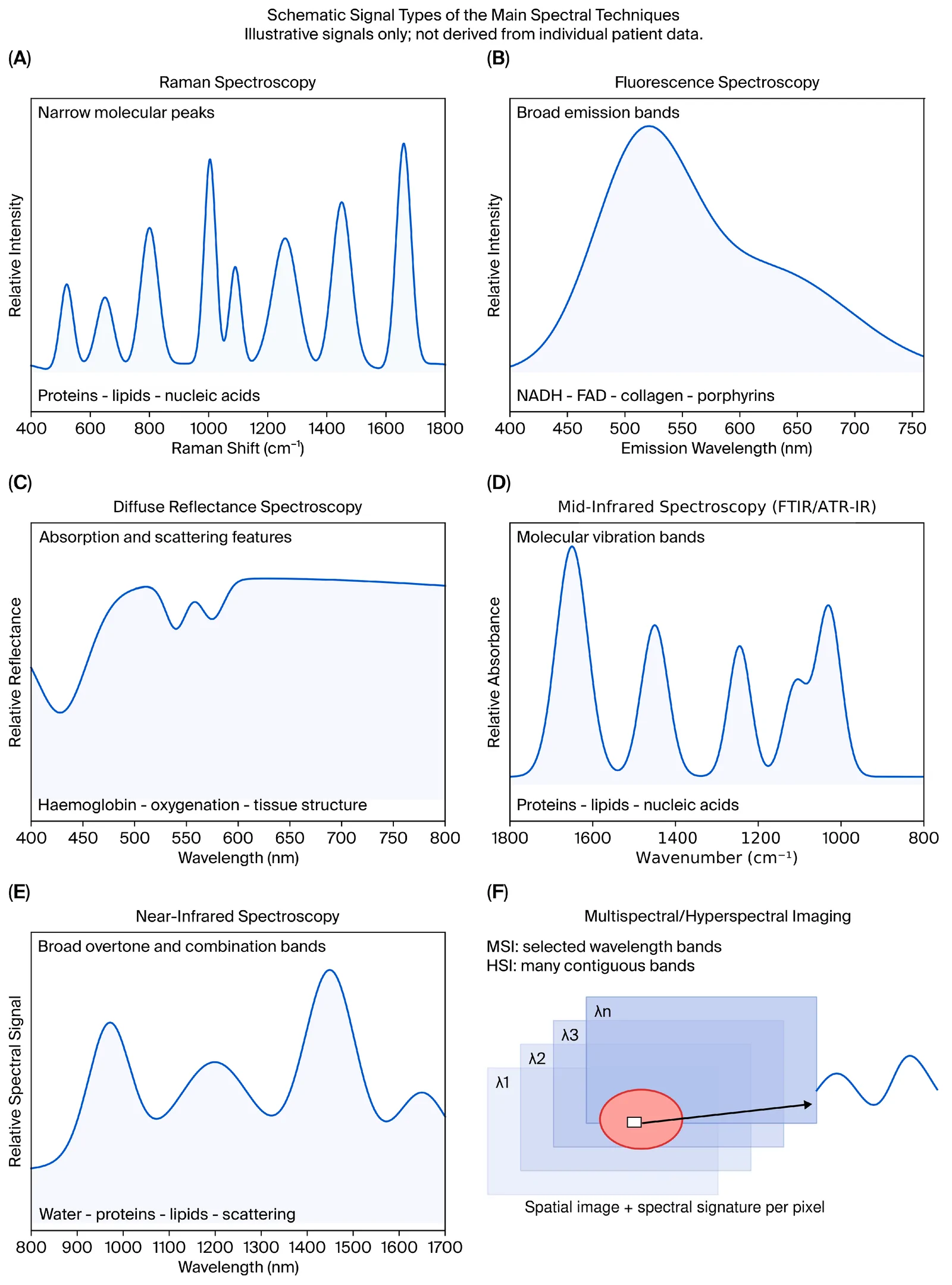
Schematic signal types of the principal spectral techniques. Mid-infrared and near-infrared signals are shown separately because they contain different spectral features. The curves are illustrative and are not derived from patient data. Abbreviations: ATR-IR, attenuated total reflectance infrared spectroscopy; FAD, flavin adenine dinucleotide; FTIR, Fourier-transform infrared spectroscopy; HSI, hyperspectral imaging; MSI, multispectral imaging; NADH, reduced nicotinamide adenine dinucleotide. Original conceptual illustration.

The included technologies capture different aspects of tissue biology, which is reflected in the signal shapes shown in Figure 3. Raman spectroscopy produces narrow peaks related to specific molecular bonds in proteins, lipids and nucleic acids. Mid-infrared spectroscopy shows molecular composition as absorption bands. Near-infrared spectroscopy produces broader, overlapping bands that are strongly influenced by water and light scattering. Cancer-related changes in cells and surrounding tissue therefore alter the overall spectral pattern rather than creating one unique cancer peak [5,13,16,45,47].

Fluorescence spectroscopy measures light emitted by naturally occurring tissue molecules linked to metabolism and structure, or by administered photosensitisers. Because several molecules contribute at once, fluorescence usually appears as broad emission bands. Diffuse reflectance measures how light is absorbed and scattered within tissue, haemoglobin, oxygenation and tissue structure therefore create a smoother curve with absorption-related dips [32,34,41,53].

None of these signals are specific to malignancy. Inflammation, bleeding, fibrosis, necrosis, cautery, tissue thickness, hydration, and changes in probe distance or angle can produce similar alterations. This helps explain why good tumour-versus-normal separation under controlled conditions does not automatically translate to high specificity during routine cystoscopy [24,42,53].

MSI and HSI apply the same principles across an image field. MSI records selected wavelength bands, whereas HSI records many adjacent bands. This provides a spectral signature for each pixel and allows spatial differences within tissue to be mapped. Spectral imaging shares the same biological sources of error and is also affected by uneven illumination, glare, viewing angle and movement [5,6,59,60]. The principal signal types are illustrated schematically in Figure 3.

### 4.3. Diagnostic Implications by Technology Family

Raman spectroscopy comes closest to the concept of an optical biopsy because it can provide molecular information from a small area without requiring a contrast agent. The main challenge is obtaining reliable spectra rapidly from all relevant parts of the bladder despite inflammation, blood, endoscopic light and treatment-related changes [18,24,28,61].

Fluorescence and reflectance spectroscopy are clinically intuitive because they can be integrated with cystoscopic inspection and biopsy targeting. Their major limitation is that inflammatory or reactive mucosa may resemble malignancy. Studies that included cystitis, scarring, or other benign abnormalities were therefore more clinically informative than studies that compared tumours only with normal tissue.

FTIR and ATR-IR studies on fixed, dried or powdered tissue show that biochemical differences can be detected, but tissue processing changes water content and tissue structure, so these findings cannot be translated directly to cystoscopic use. Studies using fresh tissue with infrared or near-infrared methods are closer to a potential role in rapid specimen assessment after TURBT, but still require standardised handling and validation in independent patients.

Multimodal systems may improve tissue characterisation by combining molecular, vascular and structural information. At the same time, each extra channel increases acquisition and calibration requirements. The available evidence does not yet show that the added complexity consistently improves clinical decisions.

The scarcity of spectral imaging remains important. Point spectroscopy can characterise a suspicious area in detail, but cystoscopy first requires wide-field detection. Real-time multispectral and hyperspectral systems may eventually bridge this gap, yet the current human bladder evidence remains very limited. Hyperspectral imaging should therefore be described as a promising future direction, not as a clinically validated modality. The potential clinical roles and current evidence position of the principal technology groups are summarised in Table 2.

### 4.4. Translational Readiness and Clinical Utility

Across modalities, the recurring problem is not a complete absence of diagnostic signal, but a lack of robust clinical proof. Few studies have evaluated outcomes that directly matter to urologists and patients, such as improved CIS detection, better biopsy selection, fewer unnecessary biopsies, more complete resection, less residual disease, or reduced recurrence.

Another important issue is the number of usable measurements. The technology may report good accuracy after excluding poor-quality spectra, but the result is clinically interpretable only when the proportion and causes of failed measurements are also reported. Latka et al. illustrated this point clearly, as a substantial proportion of patients and spectra did not contribute to the final model.

Clinical use during cystoscopy also introduces demands that are absent in specimen studies. Bladder filling and compliance alter wall tension, thickness and curvature; irrigation, urine, blood and movement can affect optical signals; and the dome, bladder neck and lateral walls are not equally easy to reach with a probe. Acquisition must also be fast enough not to disrupt the procedure. These factors should be treated as part of clinical performance rather than as engineering details alone [18,24,28,30,53].

Finally, validation should be performed at the patient level. Multiple spectra from one biopsy can help describe tissue heterogeneity, but they do not represent multiple independent patients. Randomly dividing such spectra between training and test sets can make performance appear better than it would be in new patients.

### 4.5. Comparison with Existing Reviews

This review complements broader reviews of advanced optical imaging by focusing on techniques that generate and analyse spectral information. Broader reviews are often dominated by established image-enhancement or structural techniques such as PDD, NBI, OCT and confocal endomicroscopy. The present review instead emphasises objective tissue characterisation, the relationship between point spectroscopy and spectral imaging, and the translational stage of the available human bladder evidence.

### 4.6. Strengths and Limitations

This review provides a broad and clinically oriented overview of optical spectroscopy and spectral imaging in bladder cancer. It distinguishes in vivo from ex vivo evidence, reports patient and specimen numbers, separates point-based from imaging-based approaches, and explicitly links articles that use overlapping or expanded cohorts.

The review also has limitations. The included studies were highly heterogeneous, which prevented meaningful pooling of accuracy estimates. No formal risk-of-bias assessment or meta-analysis was performed. Initial title/abstract screening and data extraction were conducted by one author. The senior author reviewed and approved the final scope and study-family framework and independently verified a targeted sample of key extracted results, but the full set of title/abstract exclusions was not independently rescreened. The protocol was not prospectively registered. Four reports, mainly older fluorescence studies, could not be retrieved, and several articles did not report all patient, specimen or validation details required for complete comparison. Finally, possible overlap remained in a small number of early research programmes and was handled conservatively.

### 4.7. Future Research Roadmap

Future studies should move from technical feasibility towards clinically focused validation. Priorities include prospective in vivo studies in realistic cystoscopy or TURBT workflows; standardised acquisition and calibration; exact matching of measured sites with histopathology; patient-level and external validation; inclusion of inflammatory and treatment-related mimics; comparison with WLC and, where relevant, BLC or NBI; and transparent reporting of failed or excluded measurements.

Artificial intelligence (AI) is likely to complement rather than replace spectral diagnostics. Recent systems have shown that AI can support lesion detection in white-light cystoscopy and real-time workflows. However, conventional images contain only visual information, whereas spectroscopy may add biochemical or optical information that is not visible under white light. AI may therefore help with signal-quality checks, identification of informative spectral patterns, and integration of spectra with images and clinical data. Future studies should compare AI-assisted white-light cystoscopy directly with AI-assisted spectral systems, particularly for difficult tasks such as CIS detection and differentiation of inflammation from malignancy [62,63,64].

Most importantly, future trials should evaluate whether the technology changes management. Relevant endpoints include CIS or high-grade lesion detection, biopsy avoidance, residual-tumour assessment, resection completeness, procedure time, need for repeat resection, and recurrence.

## 5. Conclusions

Optical spectroscopy and spectral imaging represent a promising but still developing area in bladder cancer diagnostics. The available literature suggests that these approaches can provide useful tissue information beyond conventional white-light cystoscopy, but current evidence remains heterogeneous and insufficient for routine clinical implementation. Among the spectral tissue-characterisation techniques included in this review, Raman spectroscopy currently appears to be the most translationally mature, whereas hyperspectral and other wide-field spectral imaging techniques remain underdeveloped in bladder cancer. Future studies should prioritise prospective in vivo validation, standardised acquisition, robust histopathological correlation, external patient-level validation and endpoints that demonstrate added value for real urological decision-making.

## Figures and Tables

**Table 1:** Overview of included study families. A study family comprises one or more related articles reporting the same, overlapping or expanded dataset; companion articles are shown in parentheses. A probable companion indicates likely but unconfirmed dataset overlap; a technical companion provides additional platform information without an independent bladder-specific denominator. Additional article-level characteristics and key diagnostic-performance details are provided in Supplementary Tables S1 and S2.

Main Article (Companion Article)	Setting and Tissue	Sample Size	Main Task	Key Findings
**Raman spectroscopy and Raman imaging**				
Stone et al., 2002 [12]	Ex vivo; frozen tissue	12 patients; 12 bladder samples; 196 spectra	Tumour detection and grade discrimination	89.3% of spectra were correctly classified, but validation was performed at spectrum level in only 12 samples.
Crow et al., 2004 [13] (Stone et al., 2007 [14], companion)	Ex vivo; frozen tissue	72 patients; 75 bladder samples	Tumour detection, grade and stage discrimination	Normal, cystitis and urothelial carcinoma/CIS were separated with reported sensitivities of 90% or higher and specificities of 95% or higher in spectrum-level cross-validation; grade and stage were also explored.
Crow et al., 2005 [15]	Ex vivo; frozen tissue	24 patients; 29 bladder samples	Tumour detection	Leave-one-specimen-out analysis reported 79% sensitivity, 89% specificity and 84% accuracy.
de Jong et al., 2006 [16]	Ex vivo; tissue sections	15 patients; 25 Raman maps	Tumour detection	Leave-one-patient-out analysis reported 94% sensitivity and 92% specificity.
Grimbergen et al., 2009 [17]	Ex vivo; frozen biopsies, with/without 5-ALA	73 patients without 5-ALA and 19 with 5-ALA; overlap NR; 92 and 38 biopsies; 1793 and 209 Raman spectra/measurement points, respectively	Tumour detection	Raman classification remained feasible after 5-ALA, but a model trained without 5-ALA transferred poorly to exposed biopsies without retraining; combining fluorescence and Raman improved sensitivity in the small 5-ALA subgroup.
Draga et al., 2010 [18]	In vivo; cystoscopy/TURBT	38 enrolled; 32 analysed; 83 pathology-linked sites overall, of which 63 were used in the binary tumour-detection analysis	Tumour detection and stage discrimination	Tumour detection showed 85% sensitivity and 79% specificity using site-level leave-one-out validation; stage discrimination was less accurate.
Barman et al., 2012 [19]	Ex vivo; fresh TURBT tissue	14 patients; 28 tissue sites	Tumour detection	Confocal sampling reported 85.7% sensitivity and 100% specificity in a small spectrum-level analysis.
Chen et al., 2018 [20] (Chen et al., 2020 [21], companion)	Ex vivo; bladder specimens	2018: 32 specimens, patient number NR; 2020 companion: 10 patients/42 specimens	Tumour detection and grade discrimination	Three-class accuracy was 93.1%; both articles used repeated spectra and spectrum-level validation.
Davis et al., 2018 [22]	Ex vivo; resected tissue labelled with topical SERS nanoparticles	19 consented; classification analysis based on 15 specimens (7 normal, 8 tumour)	Tumour detection	Targeted SERS imaging showed molecular contrast, but the classification analysis performed with the Raman endoscope used only 15 specimens.
Cordero et al., 2020 [23]	Ex vivo; bladder biopsies	28 patients; 67 biopsies; 42 used in principal model	Tumour detection and grade discrimination	Tumour detection accuracy was 92% and grade accuracy 84%; heterogeneous biopsies were excluded.
Stomp-Agenant et al., 2022 [24]	In vivo; cystoscopy/TURBT	66 new superficial-probe patients + 38 reused Draga patients; 216 biopsy sites comprising 156 superficial-probe sites and 60 reused non-superficial-comparator sites	Tumour detection and grade discrimination	The superficial probe achieved 90% sensitivity, 87% specificity and an AUC of 0.95; inflammation remained a confounder.
Krafft et al., 2023 [25] (Muñoz-Bolaños et al., 2024 [26], companion)	Ex vivo; frozen resectates	10 patients; 20 paired control/tumour specimens	Tissue mapping	Raman maps identified tumour, normal tissue, necrosis and other tissue/material components; no accuracy estimate was calculated.
Liu et al., 2024 [27]	In vivo; TURBT	74 diagnostic + 53 treatment-comparison patients; separate cohorts	Tumour detection and invasion discrimination	The confusion matrix yields cancer sensitivity 80.8% and specificity 90.5%; validation was performed at spectrum level rather than patient level, and the recurrence comparison was exploratory.
Latka et al., 2024 [28]	In vivo; cystoscopy/TURBT	21 enrolled; 13 analysable; 44 biopsy sites; 800 spectra, 438 retained	Tumour detection and grade discrimination	The 83% accuracy came from internal cross-validation of the retained spectra; patient grouping was not reported, and 45% of spectra and 8 of 21 patients did not contribute usable data.
**Fluorescence, reflectance, and infrared/NIR spectroscopy**				
Baert et al., 1993 [29]	Mixed in vivo/ex vivo; biopsies and cystectomy tissue	24 patients; almost 300 histology-linked biopsy sites (exact count NR)	Tumour detection	Photofrin-enhanced fluorescence ratios differed between tumour and benign tissue; no formal accuracy model was reported.
Mourant et al., 1995 [30]	In vivo; cystoscopy	10 patients; 50 sites; 82 optical measurements	Tumour detection	Same-dataset analysis showed an apparent sensitivity of 100% and specificity of 97%.
Anidjar et al., 1996 [31]	In vivo; cystoscopy	25 patients; 66 sites	Tumour/CIS detection	At 308 nm, a same-dataset cut-off separated all tumours, including CIS, from normal or inflammatory mucosa.
Koenig et al., 1998a [32] (Koenig et al., 1996 [33], companion)	In vivo; cystoscopy	1998: 75 patients/130 areas; 1996 companion: 53 patients/114 areas; 44 lesions reused	Biopsy targeting	A threshold retaining 95% of malignant lesions could have reduced non-malignant biopsies by 72%; 44 lesions were reused from the companion article.
Koenig et al., 1998b [34]	In vivo; cystoscopy	14 patients; 26 bladder sites	Tumour detection	Diffuse reflectance achieved 91% sensitivity and 60% specificity.
Lange et al., 1999 [35]	In vivo; cystoscopy	25 enrolled; spectra in 24 patients; 109 biopsy sites	Technical feasibility	The pilot reported false-negative and false-positive rates of approximately 7% and 17%, respectively, under variable dosing and timing.
Zaak et al., 2002 [36]	In vivo; cystoscopy	43 patients; 114 biopsy-correlated sites	Tumour/CIS detection	Ultraviolet autofluorescence achieved 95% sensitivity and 77% specificity; the cut-off was selected in the same dataset.
Zheng et al., 2003 [37]	Ex vivo; fresh specimens	25 patients; 52 specimens (14 benign, 38 malignant)	Tumour detection	Excitation–emission analysis identified discriminatory wavelength combinations; performance was estimated on the same dataset.
Al-Muslet et al., 2012 [38] (Ahmed et al., 2010 [39], probable companion)	Ex vivo; fixed/processed tissue	11 patients; 22 paired normal/cancer samples	Biochemical characterisation	FTIR band differences were reported in the 11-patient series; the earlier seven-patient article also reported Fourier-transform Raman findings. The publications were treated as a probable expanded series.
Aboumarzouk et al., 2015 [40]	In vivo; TURBT/biopsy	21 patients; 67 biopsy locations	Tumour detection	Benign and malignant sites showed significant spectral differences, but no classification model was validated.
Palmer et al., 2017 [41]	Ex vivo; fresh tissue	20 patients; 122 samples, including 6 CIS	CIS detection	The optical redox ratio showed an AUC of 0.83 for CIS, but multiple samples per patient were analysed independently.
Kriegmair et al., 2018 [42]	In vivo; TURBT	25 patients; 56 lesions; spectroscopy sub-study in 6 patients	Technical feasibility	The reported visual autofluorescence performance was considered contextual; the eligible spectroscopy sub-study was descriptive and included only six patients.
Kalyagina et al., 2020 [43]	In vivo; PDD/TURBT	9 patients; 25 tissue areas	Tissue characterisation	Fluorescence and backscatter differed across healthy, dysplastic/CIS and higher-grade areas; no classification model was validated.
Yousif et al., 2020 [44]	Ex vivo; fixed/dried tissue	Patient number unclear; 46 specimens (23 normal, 23 urothelial carcinoma)	Biochemical characterisation	ATR-IR showed lipid-, protein- and collagen-related differences; no diagnostic model was developed.
Bandzevičiūtė et al., 2023 [45] (Bandzevičiūtė et al., 2020 [46], technical companion)	Ex vivo; fresh tissue	54 patients; 96 samples (49 normal, 47 tumour)	Tumour detection	Sample-level analysis reported 91% sensitivity and 96–98% specificity without independent validation. The 2020 technical companion added platform information but no independent bladder denominator.
Yim et al., 2024 [47]	Ex vivo; fresh tissue	27 patients; 71 specimens; 355 spectra	Grade and stage discrimination	Very high grade- and stage-discrimination metrics were reported, but validation was performed at spectrum level.
**Multimodal and hybrid systems**				
Bovenkamp et al., 2018 [48]	Ex vivo; fresh biopsies	8 patients; 12 biopsies	Grade and stage discrimination	OCT staging accuracy was 71%, and Raman grading accuracy was 93%, in only eight patients.
Placzek et al., 2020 [49] (Schie et al., 2021 [50], companion)	Ex vivo; bladder biopsies	44 patients; 119 biopsies	Tumour detection and grade discrimination	OCT and Raman provided complementary detection and grade information; the companion article used the same cohort.
Morselli et al., 2021 [51] (Baria et al., 2019 [52], companion)	Ex vivo; fresh TURBT/TURP specimens	114 patients; 169 specimens (40 healthy, 129 UC)	Tumour detection, grade and stage discrimination	The expanded multimodal programme reported promising internal performance for tumour detection and grade or stage discrimination.
Zlobina et al., 2024 [53]	In vivo; cystoscopy/TURBT	21 patients; 13 cancer areas from 10 confirmed cases plus benign sites; total sites NR	Tumour detection	Diffuse reflectance plus visible fluorescence achieved a cross-validated AUC of 0.84 ± 0.16; adding NIR and Raman did not improve classification.
**Spectral imaging techniques**				
Demos et al., 2004 [54]	Ex vivo; resected tissue	25 patients; selected specimens, exact number NR	Tumour visualisation and margin delineation	NIR spectral images showed high tumour contrast and enabled qualitative delineation of tumour margins.
Cicchi et al., 2010 [55]	Ex vivo; fresh biopsies	5 patients; 5 healthy and 5 CIS biopsies	CIS characterisation	Healthy mucosa and CIS differed in morphology, redox-related spectra and fluorescence lifetime; no classification model was developed.
Jacobson et al., 2012 [56]	In vivo; cystoscopy	21 patients; number of sites NR	Technical feasibility	Simultaneous white-light and NIR autofluorescence imaging was technically feasible; no accuracy analysis was performed.
Pradère et al., 2018 [57]	Ex vivo; fixed specimens	14 patients; 16 specimens (15 bladder, 1 upper tract)	Grade discrimination	Spectral redox measures differed between healthy and tumour tissue and between low- and high-grade disease.
Kriegmair et al., 2020 [58]	In vivo; real-time cystoscopy	10 patients; 31 lesions, 27 malignant	Technical feasibility	Both observers recognised all malignant lesions on multiparametric images; this was a selected feasibility study, not a formal sensitivity estimate.

Abbreviations: 5-ALA, 5-aminolevulinic acid; ATR-IR, attenuated total reflectance infrared spectroscopy; AUC, area under the curve; CIS, carcinoma in situ; FTIR, Fourier-transform infrared spectroscopy; NIR, near-infrared; NR, not reported; OCT, optical coherence tomography; PDD, photodynamic diagnosis; SERS, surface-enhanced Raman scattering; TURBT, transurethral resection of bladder tumour; TURP, transurethral resection of the prostate; UC, urothelial carcinoma.

**Table 2:** Clinical roles and current positions of the principal technology groups.

Technology Group	Main Information	Possible Clinical Role	Current Evidence	Main Limitation
**Raman spectroscopy and imaging**	Molecular composition	Local tissue characterisation and mapping	Repeated ex vivo studies, several in vivo cohorts and device-development work; most developed, but not ready for routine use	Variable signal quality, probe access/contact and limited external validation
**Fluorescence and reflectance spectroscopy**	Fluorophores, blood absorption and scattering	Lesion contrast and biopsy targeting	Long in vivo history; promising but inconsistent specificity	Inflammation, bleeding and contrast-agent workflow
**Infrared and NIR spectroscopy**	Molecular absorption	Rapid ex vivo specimen characterisation	Promising ex vivo tumour, grade and stage results; early translational stage	Tissue processing and limited patient-level validation
**Multimodal and hybrid systems**	Combined molecular, vascular and structural information	Characterisation of difficult lesions	Small, mainly ex vivo programmes; proof of concept	Added complexity and uncertain incremental benefit
**Spectral imaging (MSI/HSI)**	Spatial spectral maps	Wide-field lesion detection and mucosal mapping	Limited human feasibility evidence; underdeveloped in bladder cancer	Very limited clinical HSI data and real-time workflow integration

Abbreviations: HSI, hyperspectral imaging; MSI, multispectral imaging; NIR, near-infrared.

## Data Availability

No new data were created or analysed in this study. Data sharing is not applicable to this article.

## Supplementary Materials

The following supporting information can be downloaded at https://www.mdpi.com/article/10.3390/diagnostics16162561/s1. Supplementary File S1: PRISMA-ScR checklist; Supplementary File S2: full database search strategies; Supplementary Tables S1–S3 (provided as a single Word document): Supplementary Table S1, article-level characteristics and study-family mapping; Supplementary Table S2, family-level diagnostic tasks, validation approaches, key reported results and main interpretive limitations; Supplementary Table S3, full-text exclusions and reports not retrieved.
