# Supplementary material for: Optical Spectroscopy and Spectral Imaging for Bladder Cancer Detection and Characterisation: A Scoping Review of Diagnostic Evidence and Translational Readiness
Source: Diagnostics (Basel). 2026 Aug 14;16(16):2561. doi: 10.3390/diagnostics16162561 (PMC13512009; doi:10.3390/diagnostics16162561)
Supplement: Supplementary file 1 [file diagnostics-16-02561-s001.zip › Supplementary File S1.pdf]

Non-published material. PRISMA-ScR checklist

Optical spectroscopy and spectral imaging for bladder cancer detection and characterisation: a scoping review of diagnostic evidence and translational readiness

Checklist based on the PRISMA extension for Scoping Reviews (PRISMA-ScR). Stable manuscript section references are provided because pagination may change during journal production.

Section	Item	PRISMA-ScR checklist item	Reported in
Title	1	Identify the report as a scoping review.	Title page.
Abstract	2	Provide a structured summary including background, objectives, eligibility criteria, information sources, charting methods, results and conclusions.	Structured Abstract.
Introduction	3	Describe the rationale for the review in the context of existing knowledge and explain why the review question lends itself to a scoping-review approach.	Section 1, paragraphs 1-7.
Introduction	4	State explicit review questions and objectives with reference to the key Population, Concept and Context elements.	Section 1, final two paragraphs; Section 2.3.
Methods	5	Indicate whether a protocol exists, where it can be accessed and, if registered, the registration information.	Section 2.1: no protocol was prospectively registered.
Methods	6	Specify the characteristics used to determine eligibility and explain any relevant restrictions.	Sections 2.3 and 2.4.
Methods	7	Describe all information sources and state the date of the most recent search.	Section 2.2; Supplementary File S1.
Methods	8	Present the full electronic search strategy for at least one database, including limits, so that it can be repeated.	Supplementary File S1; all three complete database strategies are provided.
Methods	9	Describe the process for selecting sources of evidence.	Section 2.5.
Methods	10	Describe the methods used for charting data and any process for obtaining or confirming data from investigators.	Section 2.6; senior-author verification described in Section 2.5.
Methods	11	List and define all variables for which data were sought and any assumptions or simplifications made.	Section 2.6; Supplementary Tables S1 and S2.
Methods	12	If critical appraisal was performed, describe the rationale, methods and use of the appraisal results (optional item).	Section 2.7: no formal instrument was applied; relevant methodological features were charted narratively.
Methods	13	Describe the methods of handling and summarising the charted data.	Section 2.7.
Results	14	Report the numbers of sources screened, assessed for eligibility and included, with reasons for exclusions at each stage, ideally using a flow diagram.	Section 3.1; Figure 1; Supplementary Table S3.
Results	15	Present characteristics of the sources of evidence and provide citations.	Section 3.1; Table 1; Supplementary Table S1.
Results	16	If critical appraisal was performed, present the appraisal results (optional item).	Sections 3.2-3.6 and 4.1-4.6; Supplementary Table S2. No formal risk-of-bias score was assigned.
Results	17	For each included source, present the relevant charted data that relate to the review questions and objectives.	Sections 3.2-3.5; Table 1; Supplementary Tables S1 and S2.
Results	18	Summarise and/or present the charting results as they relate to the review questions and objectives.	Sections 3.2-3.6; Table 2.
Discussion	19	Summarise the main results, including the concepts, themes and types of evidence available, and relate them to the review questions and objectives.	Sections 4.1-4.5.
Discussion	20	Discuss limitations of the scoping-review process.	Section 4.6.
Discussion	21	Provide a general interpretation of the results and important implications and/or next steps.	Sections 4.7 and 5.
Funding	22	Describe funding for the included sources of evidence and for the scoping review, including the role of funders.	Declarations—Funding reports the review funding. Funding of individual included articles was not systematically charted.

Note. Items 12 and 16 are optional PRISMA-ScR items. No formal critical-appraisal instrument was applied because the evidence included descriptive, mapping, feasibility and diagnostic-classification studies; instead, the manuscript reports the methodological features used for interpretation.
