# Supplementary material for: Optical Spectroscopy and Spectral Imaging for Bladder Cancer Detection and Characterisation: A Scoping Review of Diagnostic Evidence and Translational Readiness
Source: Diagnostics (Basel). 2026 Aug 14;16(16):2561. doi: 10.3390/diagnostics16162561 (PMC13512009; doi:10.3390/diagnostics16162561)
Supplement: Supplementary file 1 [file diagnostics-16-02561-s001.zip › Supplementary File S2.pdf]

Supplementary File S2. Full database search strategies

Optical spectroscopy and spectral imaging for bladder cancer detection and characterisation: a scoping review of diagnostic evidence and translational readiness

Final search date: 31 July 2026.

Search update and scope: During revision, the final search was broadened to improve sensitivity across the existing tissue-based scope, particularly for Fourier-transform infrared spectroscopy, attenuated total reflectance infrared spectroscopy, spectroscopic imaging and related terminology. The clinical question, population and direct human bladder-tissue focus were unchanged.

Limits and supplementary searching: No date or study-design filters were applied. English-language and full-text requirements were applied during study selection. Targeted backward citation searching reviewed reference lists of included articles and relevant reviews, while forward citation searching examined later articles citing key included studies. One report entered the full-text pathway through citation searching.

Database coverage and record counts

Database / platform	Coverage	Search date	Records retrieved
PubMed/MEDLINE	Database inception to 31 July 2026	31 July 2026	1063
Embase (Embase.com)	Database inception to 31 July 2026	31 July 2026	1616
Web of Science Core Collection	Database inception to 31 July 2026	31 July 2026	1659

Record-count verification. The exported files contained 1063 PubMed records, 1616 Embase records and 1659 Web of Science records, for a total of 4338 records before deduplication. After automated and manual duplicate removal, 2501 unique records remained for screening.

Presentation note. Each exact database strategy is reproduced on a separate landscape page to facilitate checking, copying and reuse.

PubMed/MEDLINE (n = 1063)

Platform: PubMed/MEDLINE | Coverage: database inception to 31 July 2026 | Search date: 31 July 2026 | **Exact search string as executed**

```
(
  "Urinary Bladder Neoplasms"[Mesh]
  OR
  (
    bladder[tiab]
    AND
    (
      cancer*[tiab]
      OR carcinoma*[tiab]
      OR neoplas*[tiab]
      OR tumor*[tiab]
      OR tumour*[tiab]
      OR lesion*[tiab]
      OR tissue*[tiab]
      OR wall*[tiab]
      OR specimen*[tiab]
      OR biops*[tiab]
      OR resectat*[tiab]
      OR mucosa*[tiab]
    )
  )
  OR "urothelial cancer*"[tiab]
  OR "urothelial carcinoma*"[tiab]
  OR "urothelial neoplasm*"[tiab]
  OR "urothelial tumor*"[tiab]
  OR "urothelial tumour*"[tiab]
  OR "transitional cell carcinoma*"[tiab]
  OR NMIBC[tiab]
  OR MIBC[tiab]
)
AND
(
  "Spectrum Analysis, Raman"[Mesh]
  OR "Spectroscopy, Near-Infrared"[Mesh]
  OR "Spectroscopy, Fourier Transform Infrared"[Mesh]
  OR "Spectrophotometry, Infrared"[Mesh]
  OR "Spectrometry, Fluorescence"[Mesh]
  OR "Hyperspectral Imaging"[Mesh]
  OR spectroscop*[tiab]
  OR spectrophotometr*[tiab]
  OR Raman[tiab]
  OR "near infrared"[tiab]
  OR "near-infrared"[tiab]
  OR NIR[tiab]
  OR NIRS[tiab]
  OR "diffuse reflectance"[tiab]
  OR "optical reflectance"[tiab]
  OR autofluorescen*[tiab]
  OR "fluorescence spectroscop*"[tiab]
  OR "fluorescence spectra*"[tiab]
  OR "spectral imag*"[tiab]
  OR multispectral[tiab]
  OR hyperspectral[tiab]
  OR FTIR[tiab]
  OR "Fourier transform infrared"[tiab]
  OR "infrared spectroscop*"[tiab]
  OR "ATR IR"[tiab]
  OR "ATR-IR"[tiab]
  OR "attenuated total reflectance"[tiab]
  OR "vibrational spectroscop*"[tiab]
  OR "chemical imag*"[tiab]
)
```

Embase (Embase.com) (n = 1616)

Platform: Embase.com | Coverage: database inception to 31 July 2026 | Search date: 31 July 2026 | **Exact search string as executed**

```
(
  'bladder tumor'/exp
  OR
  (
    bladder:ti,ab,kw
    AND
    (
      cancer*:ti,ab,kw
      OR carcinoma*:ti,ab,kw
      OR neoplas*:ti,ab,kw
      OR tumor*:ti,ab,kw
      OR tumour*:ti,ab,kw
      OR lesion*:ti,ab,kw
      OR tissue*:ti,ab,kw
      OR wall*:ti,ab,kw
      OR specimen*:ti,ab,kw
      OR biops*:ti,ab,kw
      OR resectat*:ti,ab,kw
      OR mucosa*:ti,ab,kw
    )
  )
  OR 'urothelial cancer':ti,ab,kw
  OR 'urothelial carcinoma':ti,ab,kw
  OR 'urothelial neoplasm':ti,ab,kw
  OR 'urothelial tumor':ti,ab,kw
  OR 'urothelial tumour':ti,ab,kw
  OR 'transitional cell carcinoma':ti,ab,kw
  OR nmibc:ti,ab,kw
  OR mibc:ti,ab,kw
)
AND
(
  spectroscop*:ti,ab,kw
  OR spectrophotometr*:ti,ab,kw
  OR raman:ti,ab,kw
  OR 'near infrared':ti,ab,kw
  OR 'near-infrared':ti,ab,kw
  OR nir:ti,ab,kw
  OR nirs:ti,ab,kw
  OR 'diffuse reflectance':ti,ab,kw
  OR 'optical reflectance':ti,ab,kw
  OR autofluorescen*:ti,ab,kw
  OR 'fluorescence spectroscopy':ti,ab,kw
  OR 'fluorescence spectra':ti,ab,kw
  OR 'spectral imaging':ti,ab,kw
  OR 'spectroscopic imaging':ti,ab,kw
  OR multispectral:ti,ab,kw
  OR hyperspectral:ti,ab,kw
  OR ftir:ti,ab,kw
  OR 'fourier transform infrared':ti,ab,kw
  OR 'infrared spectroscopy':ti,ab,kw
  OR 'atr ir':ti,ab,kw
  OR 'atr-ir':ti,ab,kw
  OR 'attenuated total reflectance':ti,ab,kw
  OR 'vibrational spectroscopy':ti,ab,kw
  OR 'chemical imaging':ti,ab,kw
)
```

Web of Science Core Collection (n = 1659)

Platform: Web of Science Core Collection | Coverage: database inception to 31 July 2026 | Search date: 31 July 2026 | **Exact search string as executed**

```
TS=(
  (
    (
      bladder
      AND
      (
        cancer*
        OR carcinoma*
        OR neoplas*
        OR tumor*
        OR tumour*
        OR lesion*
        OR tissue*
        OR wall*
        OR specimen*
        OR biops*
        OR resectat*
        OR mucosa*
      )
    )
    OR
    (
      urothelial
      AND
      (
        cancer*
        OR carcinoma*
        OR neoplas*
        OR tumor*
        OR tumour*
        OR tissue*
      )
    )
    OR "transitional cell carcinoma"
    OR NMIBC
    OR MIBC
  )
  AND
  (
    spectroscop*
    OR spectrophotometr*
    OR Raman
    OR "near infrared"
    OR "near-infrared"
    OR NIR
    OR NIRS
    OR "diffuse reflectance"
    OR "optical reflectance"
    OR autofluorescen*
    OR "fluorescence spectroscopy"
    OR "fluorescence spectra"
    OR "spectral imaging"
    OR "spectroscopic imaging"
    OR multispectral
    OR hyperspectral
    OR FTIR
    OR "Fourier transform infrared"
    OR "infrared spectroscopy"
    OR "ATR IR"
    OR "ATR-IR"
    OR "attenuated total reflectance"
    OR "vibrational spectroscopy"
    OR "chemical imaging"
  )
)
```
