# Supplementary material for: Optical Spectroscopy and Spectral Imaging for Bladder Cancer Detection and Characterisation: A Scoping Review of Diagnostic Evidence and Translational Readiness
Source: Diagnostics (Basel). 2026 Aug 14;16(16):2561. doi: 10.3390/diagnostics16162561 (PMC13512009; doi:10.3390/diagnostics16162561)
Supplement: Supplementary file 1 [file diagnostics-16-02561-s001.zip › Supplementary Tables S1-S3.pdf]

Supplementary Tables S1-S3

Optical spectroscopy and spectral imaging for bladder cancer detection and characterisation: a scoping review of diagnostic evidence and translational readiness

Purpose and structure. Table S1 provides article-level characteristics and links related publications through stable study-family identifiers. Table S2 summarises the principal diagnostic task, validation approach, reported result and main interpretive limitation for each study family. Table S3 lists all full-text exclusions and reports that could not be retrieved.

Definitions. A study family comprises one or more related articles reporting the same, overlapping or expanded patient/specimen dataset. The most complete report is designated the main article; technical, preliminary or interpretive reports are designated companion articles. A probable companion indicates likely but unconfirmed dataset overlap; a technical companion provides additional platform information without an independent bladder-specific denominator. Study families are report-level groupings and should not be interpreted as fully independent patient cohorts. When a later article combined a new cohort with a reused comparator, it was assigned to the new cohort's family; the reused component remained linked to its source study and was not counted again. Study-family identifiers (SF01–SF39) link Tables S1 and S2. NR indicates not reported.

Supplementary Table S1. Article-level characteristics and study-family mapping

Counts are article-specific unless a shared family denominator is explicitly stated. Technical companion articles do not contribute an additional study family.

Raman spectroscopy and Raman imaging

SF ID	Article	Role	Technology	Setting and tissue	Patients/cohort	Specimens / sites	Article contribution	Family / overlap handling
SF01	Stone et al., 2002 [12]	Main article	Raman spectroscopy	Ex vivo; frozen tissue	12 patients	12 bladder samples; 196 spectra	Tumour detection and grade discrimination	Main article; possible precursor to the later Crow programme.
SF02	Crow et al., 2004 [13]	Main article	Raman spectroscopy	Ex vivo; frozen tissue	72 patients	75 bladder samples	Tumour detection, grade and stage discrimination	
SF02	Stone et al., 2007 [14]	Companion article	Raman spectroscopy	Ex vivo; frozen tissue	Same 72-patient cohort as Crow 2004	Same 75-sample dataset as Crow 2004	Biochemical characterisation	Companion; confirmed reuse of the Crow 2004 dataset.
SF03	Crow et al., 2005 [15]	Main article	Raman spectroscopy	Ex vivo; frozen tissue	24 patients	29 bladder samples	Tumour detection	Main article; possible overlap with the same tissue bank was not reported.
SF04	de Jong et al., 2006 [16]	Main article	Raman spectroscopy	Ex vivo; tissue sections	15 patients	15 tissue sections; 25 Raman maps	Tumour detection	
SF05	Grimbergen et al., 2009 [17]	Main article	Raman spectroscopy	Ex vivo; frozen biopsies, with/without 5-ALA	73 patients without 5-ALA and 19 with 5-ALA; overlap not reported	92 and 38 biopsies; 1793 and 209 Raman spectra/measurement points, respectively	Tumour detection	Main article; overlap between the 5-ALA and non-5-ALA cohorts was not reported.
SF06	Draga et al., 2010 [18]	Main article	Raman spectroscopy	In vivo; cystoscopy/TURBT	38 enrolled; 6 excluded; 32 analysed	83 pathology-linked sites overall; 63 used in the binary tumour-detection analysis	Tumour detection and stage discrimination	Main article; reused as the comparator cohort in Stomp-Agenant 2022.
SF07	Barman et al., 2012 [19]	Main article	Raman spectroscopy	Ex vivo; fresh TURBT tissue	14 patients	28 fresh specimens	Tumour detection	
SF08	Chen et al., 2018 [20]	Main article	Raman spectroscopy	Ex vivo; bladder specimens	Primary Chen 2018 patient count NR; companion Chen 2020 reports 10 patients	32 specimens; patient number NR	Tumour detection and grade discrimination	
SF08	Chen et al., 2020 [21]	Companion article	Raman spectroscopy	Ex vivo; bladder specimens (preparation NR)	10 patients	42 specimens; 2916 spectra	Method comparison	Technical companion; exact overlap with Chen 2018 was not reported.
SF09	Davis et al., 2018 [22]	Main article	SERS imaging	Ex vivo; resected tissue labelled with topical SERS nanoparticles	19 patients consented; patient count contributing to the 15-sample classifier NR	15 classification specimens (7 non-tumour, 8 tumour)	Tumour detection	Main article; exogenous molecular contrast.
SF10	Cordero et al., 2020 [23]	Main article	Raman spectroscopy	Ex vivo; bladder biopsies	28 patients	67 biopsies; 42 used in the principal model	Tumour detection and grade discrimination	
SF11	Stomp-Agenant et al., 2022 [24]	Main article	Raman spectroscopy	In vivo; cystoscopy/TURBT	66 new patients + 38 reused Draga patients	216 biopsy sites: 156 superficial-probe sites and 60 reused nonsuperficial-comparator sites	Tumour detection and grade discrimination	Main article; includes the reused Draga comparator cohort.
SF12	Krafft et al., 2023 [25]	Main article	Raman imaging	Ex vivo; frozen resectates	10 patients	20 paired control/tumour specimens	Tissue mapping	
SF12	Muñoz-Bolaños et al., 2024 [26]	Companion article	Raman imaging	Ex vivo; frozen resectates	Same 10-patient cohort as Krafft 2023	Same 20-specimen dataset; 13/19 usable maps in detailed results	Technical platform development	Technical companion; same resectate programme.

SF ID	Article	Role	Technology	Setting and tissue	Patients/cohort	Specimens / sites	Article contribution	Family / overlap handling
SF13	Liu et al., 2024 [27]	Main article	Raman spectroscopy	In vivo; TURBT	74 diagnostic + 53 treatment-comparison patients	Diagnostic cohort: exact site/biopsy count NR	Tumour detection and invasion discrimination	Main article; contains separate diagnostic and treatment-comparison cohorts.
SF14	Latka et al., 2024 [28]	Main article	Raman spectroscopy	In vivo; cystoscopy/TURBT	21 enrolled; 13 analysable	44 biopsy sites; 800 spectra, 438 retained	Tumour detection and grade discrimination	

Fluorescence, reflectance, and infrared/NIR spectroscopy

SF ID	Article	Role	Technology	Setting and tissue	Patients/cohort	Specimens / sites	Article contribution	Family / overlap handling
SF15	Baert et al., 1993 [29]	Main article	Photofrin-enhanced fluorescence spectroscopy	Mixed in vivo/ex vivo; biopsies and cystectomy tissue	24 patients (21 in vivo, 3 ex vivo)	Almost 300 histology-linked biopsy sites; exact count NR	Tumour detection	
SF16	Mourant et al., 1995 [30]	Main article	Elastic-scattering spectroscopy	In vivo; cystoscopy	10 patients	50 biopsy sites; 82 optical measurements	Tumour detection	
SF17	Anidjar et al., 1996 [31]	Main article	Autofluorescence spectroscopy	In vivo; cystoscopy	25 patients	66 biopsy sites	Tumour/CIS detection	
SF18	Koenig et al., 1998 [32]	Main article	Autofluorescence spectroscopy	In vivo; cystoscopy	75 patients	130 bladder areas	Biopsy targeting	Main article; expanded series.
SF18	Koenig et al., 1996 [33]	Companion article	Autofluorescence spectroscopy	In vivo; cystoscopy	53 patients	114 bladder areas; 44 lesions reused in 1998 article	Tumour detection	Companion; 44 lesions were reused in the 1998 article.
SF19	Koenig et al., 1998 [34]	Main article	Diffuse reflectance spectroscopy	In vivo; cystoscopy	14 patients	26 biopsy sites	Tumour detection	Main article; separate diffuse-reflectance cohort.
SF20	Lange et al., 1999 [35]	Main article	5-ALA/PpIX fluorescence spectroscopy	In vivo; cystoscopy	25 enrolled; 24 measured	109 biopsy sites	Technical feasibility	Main article; contrast-agent optimisation study.
SF21	Zaak et al., 2002 [36]	Main article	Autofluorescence spectroscopy	In vivo; cystoscopy	43 patients	114 biopsies: 21 malignant, 93 nonmalignant	Tumour/CIS detection	
SF22	Zheng et al., 2003 [37]	Main article	Autofluorescence spectroscopy	Ex vivo; fresh specimens	25 patients	52 fresh specimens (14 non-malignant, 38 malignant)	Tumour detection	
SF23	Al-Muslet et al., 2012 [38]	Main article	FTIR spectroscopy	Ex vivo; fixed/processed tissue	11 patients	22 paired non-tumour/tumour samples	Biochemical characterisation	Main article; probable expanded series.
SF23	Ahmed et al., 2010 [39]	Companion article	FTIR and Raman spectroscopy	Ex vivo; fixed/processed tissue	7 patients	14 paired non-tumour/tumour samples	Biochemical characterisation	Probable companion/precursor to Al-Muslet 2012.
SF24	Aboumarzouk et al., 2015 [40]	Main article	Autofluorescence spectroscopy	In vivo; TURBT/biopsy	21 patients	67 biopsy sites	Tumour detection	
SF25	Palmer et al., 2017 [41]	Main article	Autofluorescence spectroscopy	Ex vivo; fresh tissue	20 patients	122 fresh samples (33 non-tumour, 89 tumour)	CIS detection	
SF26	Kriegmair et al., 2018 [42]	Main article	Autofluorescence imaging with spectroscopy substudy	In vivo; TURBT	25 patients	56 lesions; spectroscopy substudy in 6 patients	Technical feasibility	Main article; visual AFI results are contextual, while the spectroscopy substudy is eligible.
SF27	Kalyagina et al., 2020 [43]	Main article	Fluorescence and backscatter spectroscopy	In vivo; PDD/TURBT	9 patients	25 tissue areas; exact biopsy count NR	Tissue characterisation	Main article; exogenous 5-ALA was central to the method.
SF28	Yousif et al., 2020 [44]	Main article	ATR-IR spectroscopy	Ex vivo; fixed/dried tissue	Patient number NR	46 specimens (23 non-tumour, 23 UC)	Biochemical characterisation	Main article; patient denominator unclear.
SF29	Bandzevičiūtė et al., 2023 [45]	Main article	ATR-IR spectroscopy	Ex vivo; fresh tissue	54 patients	96 samples (49 non-tumour, 47 tumour)	Tumour detection	
SF29	Bandzevičiūtė et al., 2020 [46]	Companion article	Fibre-based IR spectroscopy	Ex vivo; fresh multi-organ tissue	Multi-organ cohort; bladder-specific number NR	Bladder-specific specimen count NR	Technical platform development	Technical companion; no independent bladder denominator.
SF30	Yim et al., 2024 [47]	Main article	NIR spectroscopy	Ex vivo; fresh tissue	27 patients	71 specimens; 355 spectra	Grade and stage discrimination	

Multimodal and hybrid systems

SF ID	Article	Role	Technology	Setting and tissue	Patients/cohort	Specimens / sites	Article contribution	Family / overlap handling
SF31	Bovenkamp et al., 2018 [48]	Main article	OCT–Raman	Ex vivo; fresh biopsies	8 patients	12 biopsies (7 low-grade, 5 high-grade)	Grade and stage discrimination	
SF32	Placzek et al., 2020 [49]	Main article	OCT–Raman	Ex vivo; bladder biopsies	44 patients	119 biopsies	Tumour detection and grade discrimination	
SF32	Schie et al., 2021 [50]	Companion article	OCT–Raman	Ex vivo; bladder biopsies	Same 44-patient cohort as Placzek 2020	Same 119-biopsy cohort as Placzek 2020	Morpho-molecular interpretation	Companion; same 44-patient/119-biopsy cohort.
SF33	Morselli et al., 2021 [51]	Main article	Multimodal fibre spectroscopy	Ex vivo; fresh TURBT/TURP specimens	114 patients (78 TURBT, 36 TURP)	169 fresh specimens (40 non-tumour, 129 UC)	Tumour detection, grade and stage discrimination	Main article; expanded programme.
SF33	Baria et al., 2019 [52]	Companion article	Multimodal fibre spectroscopy	Ex vivo; fresh TURBT/TURP specimens	Preliminary subset of the expanded 114-patient programme; exact subset NR	Preliminary subset; exact specimen count NR	Preliminary clinical results	Companion; preliminary results from the later expanded programme.
SF34	Zlobina et al., 2024 [53]	Main article	Multimodal spectroscopy	In vivo; cystoscopy/TURBT	21 patients	13 cancer areas from 10 confirmed cases plus benign sites; total sites NR	Tumour detection	

Spectral imaging techniques

SF ID	Article	Role	Technology	Setting and tissue	Patients/cohort	Specimens / sites	Article contribution	Family / overlap handling
SF35	Demos et al., 2004 [54]	Main article	NIR spectral imaging	Ex vivo; resected tissue	25 patients	Selected specimens; exact number NR	Tumour visualisation and margin delineation	
SF36	Cicchi et al., 2010 [55]	Main article	Two-photon spectral imaging	Ex vivo; fresh biopsies	5 patients	10 biopsies (5 non-tumour, 5 CIS)	CIS characterisation	
SF37	Jacobson et al., 2012 [56]	Main article	NIR autofluorescence imaging	In vivo; cystoscopy	21 patients	Number of sites NR	Technical feasibility	
SF38	Pradère et al., 2018 [57]	Main article	Two-photon spectral imaging	Ex vivo; fixed specimens	14 patients	16 specimens (15 bladder, 1 upper tract)	Grade discrimination	Main article; includes one upper-tract specimen.
SF39	Kriegmair et al., 2020 [58]	Main article	Multispectral imaging	In vivo; real-time cystoscopy	10 patients	31 lesions (27 malignant)	Technical feasibility	

Abbreviations: 5-ALA, 5-aminolevulinic acid; ATR-IR, attenuated total reflectance infrared spectroscopy; CIS, carcinoma in situ; FTIR, Fourier-transform infrared spectroscopy; NIR, near-infrared; NR, not reported; OCT, optical coherence tomography; PDD, photodynamic diagnosis; PpIX, protoporphyrin IX; SERS, surface-enhanced Raman scattering; TURBT, transurethral resection of bladder tumour; UC, urothelial carcinoma.

Supplementary Table S2. Family-level diagnostic tasks, validation and reported performance

Histopathology was the reference standard unless otherwise stated. Values are reported as presented in the source articles and were not pooled. Same-dataset and spectrum-level estimates should not be interpreted as external patient-level validation.

Raman spectroscopy and Raman imaging

Study family	Main task	Analysis and validation	Key reported result	Main interpretive limitation
SF01 — Stone et al., 2002 [12]	Tumour detection and grade discrimination	PCA–LDA; leave-one-spectrum-out cross-validation	Overall, 89.3% of spectra were correctly classified; class sensitivities/specificities ranged from 78–98%/96–99%.	Spectrum-level validation in only 12 samples; possible precursor to the later Crow programme.
SF02 — Crow et al., 2004 [13] (Stone et al., 2007 [14], companion)	Tumour detection, grade and stage discrimination	PCA–LDA; leave-one-spectrum-out cross-validation	Normal, cystitis and malignant tissue showed reported sensitivities of 90–95% and specificities of 95–98%; grade and stage were also explored.	Repeated spectra from the same samples; Stone 2007 reused the dataset.
SF03 — Crow et al., 2005 [15]	Tumour detection	PCA–LDA; leave-one-specimen-out cross-validation	Sensitivity 79%, specificity 89% and accuracy 84% for tumour detection.	Small frozen-tissue cohort; possible tissue-bank overlap was not reported.
SF04 — de Jong et al., 2006 [16]	Tumour detection	HCA/LDA; leave-one-patient-out cross-validation	Leave-one-patient-out sensitivity 94%, specificity 92% and accuracy 93%; apparent same-data AUC 0.99.	Only 15 patients and tissue-section measurements.
SF05 — Grimbergen et al., 2009 [17]	Tumour detection	PCA–LDA; leave-one-sample-out cross-validation	Without 5-ALA: sensitivity 88.0%, specificity 80.1%; applying that model to 5-ALA-exposed biopsies without retraining yielded 42.6% sensitivity and 71.1% specificity. After retraining, fluorescence plus Raman achieved 100% sensitivity and 80.8% specificity in the small 5-ALA cohort.	Two cohorts; overlap was not reported. Model performance changed after 5-ALA exposure, and validation remained sample based.
SF06 — Draga et al., 2010 [18]	Tumour detection and stage discrimination	PCA–LDA; site-level leave-one-out validation	Tumour detection sensitivity was 85% and specificity 79% in 63 pathology-linked sites; three-class stage performance was lower.	Only 32 patients were analysed; 83 sites were available overall, but the binary analysis used 63; stage discrimination was limited.
SF07 — Barman et al., 2012 [19]	Tumour detection	PCA/logistic regression; leave-one-spectrum-out cross-validation	Confocal probe: sensitivity 85.7%, specificity 100%, accuracy 92.9% and AUC 0.91.	Only 14 patients; spectrum-level validation.
SF08 — Chen et al., 2018 [20] (Chen et al., 2020 [21], companion)	Tumour detection and grade discrimination	PCA–ANN; 10-fold cross-validation; patient/specimen grouping not reported	Overall three-class accuracy 93.1%; class sensitivities 88.5–97.5% and specificities 95.1–98.0%.	Repeated spectra and unclear grouping create a high risk of optimistic performance; exact overlap with the companion is unclear.
SF09 — Davis et al., 2018 [22]	Tumour detection	LDA/ROC analysis; leave-one-sample-out cross-validation	Reported AUCs 0.93–0.95; leave-one-out classification error 20–33% depending on normalisation.	Only 15 classification specimens; exogenous nanoparticles and ex vivo workflow.
SF10 — Cordero et al., 2020 [23]	Tumour detection and grade discrimination	Hierarchical LDA; biopsy-level k-fold cross-validation	Tumour detection: sensitivity 92%, specificity 93%, accuracy 92%; grade: 85%, 83% and 84%.	Nineteen biopsies were excluded; the principal model used 42 biopsies.
SF11 — Stomp-Agenant et al., 2022 [24]	Tumour detection and grade discrimination	PCA–LDA; leave-one-patient-out cross-validation	Superficial probe: sensitivity 90%, specificity 87% and AUC 0.95.	Superficial-probe metrics were derived from the 66-patient/156-site cohort; 60 comparator sites reused the Draga cohort, and inflammation remained a confounder.
SF12 — Krafft et al., 2023 [25] (Muñoz-Bolaños et al., 2024 [26], companion)	Tissue mapping	Unsupervised spectral unmixing and clustering; no diagnostic classifier	Tumour, non-tumour, necrosis and other tissue/material signatures were mapped; no accuracy estimate was calculated.	Ten-patient ex vivo mapping study; 13/20 specimens in the abstract versus 13/19 usable datasets in detailed results.
SF13 — Liu et al., 2024 [27]	Tumour detection and invasion discrimination	Discriminant analysis; leave-one-spectrum-out cross-validation	From the confusion matrix, tumour sensitivity was 80.8% and specificity 90.5%; invasion sensitivity/specificity were 83.3%/87.3%.	Repeated spectra within patients; no patient-level validation. Treatment findings were exploratory and do not isolate the effect of Raman diagnosis.
SF14 — Latka et al., 2024 [28]	Tumour detection and grade discrimination	PCA–LDA; internal 10-fold cross-validation of retained spectra; patient grouping not reported	Tumour detection accuracy 83% (sensitivity 83%, specificity 75%); grade accuracy 75%.	Only 13/21 patients and 438/800 spectra were analysable; whether spectra were grouped by patient during validation was not reported.

Fluorescence, reflectance, and infrared/NIR spectroscopy

Study family	Main task	Analysis and validation	Key reported result	Main interpretive limitation
SF15 — Baert et al., 1993 [29]	Tumour detection	Within-patient spectral ratios; descriptive analysis only	Tumour-selective Photofrin fluorescence ratios were demonstrated across >800 spectra and almost 300 biopsy-linked sites; no formal accuracy model.	Contrast-agent study with incomplete exact site denominator and no classifier.
SF16 — Mourant et al., 1995 [30]	Tumour detection	Single spectral-slope rule developed and tested in the same cohort	Apparent sensitivity 100% and specificity 97% in the development cohort.	Ten patients and no independent validation.
SF17 — Anidjar et al., 1996 [31]	Tumour/CIS detection	Single ratio cut-off developed and tested in the same cohort	The selected 308-nm cut-off classified all tumours, including CIS, in the analysed cohort.	Small cohort and same-dataset threshold.
SF18 — Koenig et al., 1998 [32] (Koenig et al., 1996 [33], companion)	Biopsy targeting	Ratio threshold/percentile analysis; no independent validation	A threshold retaining 95% of malignant lesions could have reduced non-malignant biopsies by 72%.	Partial cohort reuse and no independent clinical impact validation.
SF19 — Koenig et al., 1998 [34]	Tumour detection	Optical-parameter model and threshold developed in the same cohort	Sensitivity 91%, specificity 60%, PPV 63% and NPV 90%.	Fourteen patients; inflammatory and malignant lesions overlapped.
SF20 — Lange et al., 1999 [35]	Technical feasibility	Descriptive histology–fluorescence correlation; no validated classifier	Approximate false-negative and false-positive rates were 7% and 17%, respectively.	Dose/timing optimisation rather than a validated diagnostic study.
SF21 — Zaak et al., 2002 [36]	Tumour/CIS detection	Spectral-ratio threshold selected and tested in the same cohort	Autofluorescence sensitivity 95% and specificity 77%; 5-ALA fluorescence sensitivity 90% and specificity 61%.	Threshold selected in the same dataset.
SF22 — Zheng et al., 2003 [37]	Tumour detection	Spectral ratios/linear boundary developed and tested in the same cohort	Single-ratio performance reached 95–97% sensitivity and 92–93% specificity; a combined same-dataset rule reached apparent 100%/100%.	All thresholds and the decision boundary were developed and tested in the same dataset.
SF23 — Al-Muslet et al., 2012 [38] (Ahmed et al., 2010 [39], companion)	Biochemical characterisation	Descriptive FTIR/Raman comparison; no classifier	Cancer-associated changes were reported in protein, lipid and nucleic-acid bands; no diagnostic performance estimate.	Fixed/dried processed tissue; probable overlap between the two articles.
SF24 — Aboumarzouk et al., 2015 [40]	Tumour detection	Group comparisons only; no diagnostic classifier	Benign and malignant sites differed in spectral area, mean wavelength and fluorescence ratio; no validated threshold.	Small in vivo cohort and no diagnostic decision rule.
SF25 — Palmer et al., 2017 [41]	CIS detection	Exploratory ROC analysis at sample level; no patient-grouped validation	Optical redox ratio AUC 0.64 for all cancer and 0.83 for CIS.	Only six CIS samples; multiple samples per patient were analysed independently.
SF26 — Kriegmair et al., 2018 [42]	Technical feasibility	Visual observer analysis; descriptive spectroscopy substudy	Visual AFI sensitivity 96.7% and specificity 53.8%; the eligible spectroscopy substudy included only six patients and was descriptive.	Visual imaging dominated; the eligible spectroscopy component was very small.
SF27 — Kalyagina et al., 2020 [43]	Tissue characterisation	Descriptive fluorescence/backscatter indices; no classifier	Fluorescence and backscatter differed across healthy, dysplastic/CIS and higher-grade areas; no validated accuracy estimate.	Nine patients; exogenous 5-ALA and no validated model.
SF28 — Yousif et al., 2020 [44]	Biochemical characterisation	Descriptive ATR-IR band-ratio analysis; no classifier	Lipid-, protein- and collagen-related band differences were reported; no diagnostic model.	Patient denominator unclear; fixed/dried tissue.
SF29 — Bandzevičiūtė et al., 2023 [45] (Bandzevičiūtė et al., 2020 [46], companion)	Tumour detection	HCA/PCA on the full dataset; no independent holdout cohort	Sensitivity 91%; specificity 98% by HCA and 96% by PCA.	No independent validation; the technical companion had no separate bladder denominator.
SF30 — Yim et al., 2024 [47]	Grade and stage discrimination	Stacked machine-learning models; validation used repeated spectra and grouping was unclear	Low- vs high-grade: sensitivity 97%, specificity 99%, AUC 0.995 in the detailed results table (0.997 reported elsewhere in the article); stage classification: sensitivity 97%, specificity 92%, AUC 0.996.	Multiple spectra per specimen and unclear patient grouping.

Multimodal and hybrid systems

Study family	Main task	Analysis and validation	Key reported result	Main interpretive limitation
SF31 — Bovenkamp et al., 2018 [48]	Grade and stage discrimination	k-nearest-neighbour models; 5-fold cross-validation	OCT stage accuracy 71%; Raman grade sensitivity 99%, specificity 87% and accuracy 93%.	Only eight patients.
SF32 — Placzek et al., 2020 [49] (Schie et al., 2021 [50], companion)	Tumour detection and grade discrimination	OCT texture and hierarchical Raman models; biopsy-partitioned k-fold cross-validation	OCT detection sensitivity 78%, specificity 69%, accuracy 73.4%; Raman grade sensitivity 81%, specificity 68%, accuracy 77%.	Single-centre ex vivo cohort; companion article reused the same 44 patients/119 biopsies.
SF33 — Morselli et al., 2021 [51] (Baria et al., 2019 [52], companion)	Tumour detection, grade and stage discrimination	PCA–LDA/QDA; 5-fold cross-validation; patient grouping not reported	Fivefold-CV AUCs: tumour detection 0.88; grade 0.78; stage-pair comparisons 0.74–0.80.	Specimen-level internal validation, patient grouping unclear; stage denominators differed between abstract and detailed results.
SF34 — Zlobina et al., 2024 [53]	Tumour detection	Logistic regression; internal cross-validation; grouping not reported	Diffuse reflectance plus visible fluorescence: sensitivity 78% ± 36, specificity 91% ± 11 and AUC 0.84 ± 0.16.	Only 10 histologically confirmed cancer cases; inflammation and probe geometry affected classification.

Spectral imaging techniques

Study family	Main task	Analysis and validation	Key reported result	Main interpretive limitation
SF35 — Demos et al., 2004 [54]	Tumour visualisation and margin delineation	ROI-based image comparison with histopathology; no diagnostic classifier	NIR spectral images showed high tumour contrast and qualitative margin delineation; no sensitivity/specificity estimate.	Selected ex vivo specimens; exact specimen denominator and formal accuracy were not reported.
SF36 — Cicchi et al., 2010 [55]	CIS characterisation	Morphological/spectral group comparisons; no diagnostic classifier	Healthy mucosa and CIS differed in morphology, redox-related spectra and fluorescence lifetime; no classifier.	Five paired patient sets and no diagnostic validation.
SF37 — Jacobson et al., 2012 [56]	Technical feasibility	Qualitative paired-image feasibility assessment; no accuracy analysis	Simultaneous white-light and NIR autofluorescence imaging was feasible in 21 patients; no accuracy estimate.	Technical feasibility only; number of measured sites was not reported.
SF38 — Pradère et al., 2018 [57]	Grade discrimination	ROI-level spectral/statistical comparisons; no diagnostic classifier	Redox measures differed between non-tumour and tumour tissue and between low- and high-grade disease; no classifier.	Fourteen patients, fixed specimens, repeated ROIs and one upper-tract specimen.
SF39 — Kriegmair et al., 2020 [58]	Technical feasibility	Two-observer image rating; no formal diagnostic-accuracy analysis	Both observers recognised all 27 malignant lesions on multiparametric images; this was not a formal sensitivity estimate.	Selected 10-patient feasibility study with post-procedure observer ratings.

Abbreviations: ANN, artificial neural network; AUC, area under the curve; CIS, carcinoma in situ; CV, cross-validation; HCA, hierarchical cluster analysis; LDA, linear discriminant analysis; OCT, optical coherence tomography; PCA, principal component analysis; PPV, positive predictive value; NPV, negative predictive value; QDA, quadratic discriminant analysis; ROC, receiver operating characteristic.

Supplementary Table S3. Full-text exclusions and reports not retrieved

One primary reason is reported for each full-text exclusion. The PRISMA categories and counts correspond to Figure 1.

Full-text excluded

Status	Article	Primary reason	PRISMA category	Identifier
Full-text excluded	Laser-induced autofluorescence diagnosis of tumors (1997)	Duplicate/overlapping report without additional extractable bladder evidence.	Duplicate report without additional evidence	DOI: 10.1088/0031-8949/1997/t72/012
Full-text excluded	[Early diagnosis of bladder cancer by special fluorescence spectrum: an in vitro experiment]. (2007)	Non-English full text.	Non-English full text	PMID: 18067829
Full-text excluded	[Fluorescence detection of chlorine E6 based photosensitizer in urinary bladder tumors]. (2007)	Non-English full text.	Non-English full text	PMID: 17578199
Full-text excluded	[Superficial bladder cancer detection using diffuse reflectance spectral ratio R540/R575 of oxygenated hemoglobin bands]. (2008)	Non-English full text.	Non-English full text	DOI: 10.3964/j.issn.1000-0593(2008)11-2721-05
Full-text excluded	Photodynamic Diagnosis And Fluorescence Spectroscopy In Superficial Bladder Cancer (2009)	Non-English full text.	Non-English full text	NR
Full-text excluded	[Study on bladder cancer tissues with Raman spectroscopy]. (2012)	Non-English full text.	Non-English full text	DOI: 10.3964/j.issn.1000-0593(2012)01-0123-04
Full-text excluded	High-precision bladder cancer diagnosis method: 2D Raman spectrum figures based on maintenance technology combined with automatic weighted feature fusion network. (2023)	Secondary AI reanalysis of an existing Raman dataset; no new human tissue acquisition or cohort.	Secondary analysis without a new cohort	DOI: 10.1016/j.aca.2023.341908
Full-text excluded	Time-resolved spectrofluorometer for clinical tissue characterization during endoscopy (1999)	Instrumentation paper without original human bladder diagnostic data.	Wrong concept / no eligible tissue assessment	DOI: 10.1063/1.1150038
Full-text excluded	Autofluorescence imaging to optimize 5-ALA-induced fluorescence endoscopy of bladder carcinoma. (2001)	RGB/image-enhancement study without wavelength-resolved spectral analysis.	Wrong concept / no eligible tissue assessment	DOI: 10.1016/s0090-4295(01)01222-5
Full-text excluded	Fluorescent diagnosis of urinary bladder cancer - A comparison of two diagnostic modalities (2004)	Visual colour-coded fluorescence/PDD comparison without wavelength-resolved spectral analysis.	Wrong concept / no eligible tissue assessment	DOI: 10.1016/s1572-1000(04)00006-7
Full-text excluded	Use of picosecond Kerr-gated Raman spectroscopy to suppress signals from both surface and deep layers in bladder and prostate tissue - art. no. 044006 (2005)	Technical Raman depth-profiling study without a diagnostic classification or clinical tissue-characterisation endpoint.	Wrong concept / no eligible tissue assessment	DOI: 10.1117/1.1991848
Full-text excluded	Evaluation of autofluorescence and photodynamic diagnosis in assessment of bladder lesions. (2020)	Autofluorescence/PDD image-enhancement study without wavelength-resolved spectral analysis.	Wrong concept / no eligible tissue assessment	DOI: 10.1016/j.pdpdt.2020.101719
Full-text excluded	Tissue characterization using high wave number Raman spectroscopy - art. no. 031116 (2005)	Mixed-organ thin-section technical comparison without a separately extractable bladder-specific diagnostic result.	Wrong specimen or processed-section context	DOI: 10.1117/1.1922307
Full-text excluded	HPLC assisted Raman spectroscopic studies on bladder cancer (2015)	Processed-section confocal Raman/HPLC molecular pathology, outside the intact-specimen/endoscopic context.	Wrong specimen or processed-section context	DOI: 10.1088/1612-2011/12/4/045701
Full-text excluded	Raman spectroscopy of luminal subtype and basal subtype muscle invasive bladder cancer (2019)	Processed-section SERS molecular-subtype classification, outside the intact-specimen/endoscopic context.	Wrong specimen or processed-section context	NR
Full-text excluded	Classification of tissue biopsies by Raman spectroscopy guided by quantitative phase imaging and its application to bladder cancer. (2022)	Quantitative-phase-guided Raman analysis of sliced tissue sections, outside the eligible context.	Wrong specimen or processed-section context	DOI: 10.1002/jbio.202200009
Full-text excluded	Toward a SERS Diagnostic Tool for Discrimination between Cancerous and Normal Bladder Tissues via Analysis of the Extracellular Fluid. (2022)	Tissue-derived extracellular fluid rather than direct assessment of bladder tissue.	Wrong specimen or processed-section context	DOI: 10.1021/acsomega.2c00058

Report not retrieved

Status	Article	Primary reason	PRISMA category	Identifier
Report not retrieved	Fluorescence imaging of bladder cancer. (1994)	Full text not retrieved after targeted retrieval attempts.	Report not retrieved	PMID: 7793348
Report not retrieved	In vivo fluorescence detection of human bladder carcinoma without sensitizing agents. (1994)	Full text not retrieved after targeted retrieval attempts.	Report not retrieved	DOI: 10.1080/01952307.1994.11735929
Report not retrieved	Detection of papillary tumors of the urinary bladder using fluorescence spectroscopy (2000)	Full text not retrieved after targeted retrieval attempts.	Report not retrieved	NR
Report not retrieved	Detection of bladder urothelial carcinoma using in vivo noncontact, ultraviolet excited autofluorescence measurements converted into simple color coded images: a feasibility study. (2013)	Full text not retrieved after targeted retrieval attempts.	Report not retrieved	DOI: 10.1016/j.juro.2013.01.100
